# FATS regulates polyamine biosynthesis by promoting ODC degradation in an ERβ-dependent manner in non-small-cell lung cancer

**DOI:** 10.1038/s41419-020-03052-1

**Published:** 2020-10-09

**Authors:** Li Qiu, Linfei Hu, Huijuan Wang, Jinling Li, Xianhui Ruan, Bingsheng Sun, Jingtai Zhi, Xiangqian Zheng, Lin Gu, Ming Gao, Pengzhou Kong, Jun Zhang

**Affiliations:** 1 Department of Cancer Cell Biology, Tianjin’s Key Laboratory of Cancer Prevention and Therapy, National Clinical Research Center for Cancer, Tianjin Medical University Cancer Institute and Hospital, Tianjin, 300060 P. R. China; 2 Department of Thyroid and Neck Tumor, Tianjin Medical University Cancer Institute and Hospital, National Clinical Research Center for Cancer, Tianjin’s Clinical Research Center for Cancer, Key Laboratory of Cancer Prevention and Therapy, Tianjin, 300060 P. R. China; 3 Department of Pharmacy, Chongqing Jiu Long Po People’s Hospital, Chongqing, 400000 P. R. China; 4 Department of Lung Cancer, Tianjin Lung Cancer Center, Tianjin’s Key Laboratory of Cancer Prevention and Therapy, Tianjin Medical University Cancer Institute and Hospital, Tianjin, 300060 P. R. China; 5 Department of Breast Cancer, Key Laboratory of Breast Cancer Prevention and Therapy, National Clinical Research Center for Cancer, Tianjin’s Clinical Research Center for Cancer, Tianjin Medical University Cancer Institute and Hospital, Tianjin, 300060 P. R. China; 6 Department of Pathology & Shanxi Key Laboratory of Carcinogenesis and Translational Research of Esophageal Cancer, Shanxi Medical University, Taiyuan, Shanxi 030001 P. R. China

**Keywords:** Macroautophagy, Apoptosis

## Abstract

Polyamine biosynthesis is an essential metabolic pathway for cell growth and differentiation in non-small-cell lung cancer (NSCLC). Fragile-site associated tumour suppressor (FATS) is a novel gene involved in cancer. The results of our previous study showed that FATS-mediated polyubiquitination of p53 promotes the activation of p53 in response to DNA damage; however, little is known about the role of FATS in metabolic reprogramming in NSCLC. In the present study, FATS was observed to be significantly downregulated in NSCLC tissues compared with paired adjacent normal tissues and was associated with the survival of NSCLC patients. We further showed that the presence of the tumour suppressor FATS in NSCLC cells led to apoptosis by inducing pro-death autophagy. In addition, FATS was shown to function as a suppressor of polyamine biosynthesis by inhibiting ornithine decarboxylase (ODC) at the protein and mRNA levels, which was partially dependent on oestrogen receptor (ER). Furthermore, FATS was observed to bind to ERβ and translocate to the cytosol, leading to ODC degradation. The findings of our study demonstrate that FATS plays important roles in polyamine metabolism in NSCLC and provides a new perspective for NSCLC progression.

## Introduction

To maintain their growth, cancer cells need to constantly obtain nutrients, including amino acids, nucleic acids and fatty acids, through external intake or self-synthesis^1^ and require that metabolism inside cells be maintained in a balanced state^2^. Introducing an additional substance or an abnormal supply of nutrients will disturb this balance, causing an altered growth state of cells and leading to apoptosis or autophagy^3^. This process can also affect the progression of non-small-cell lung cancer (NSCLC)^4,5^.

Apoptosis is defined as a morphologically distinct form of programmed cell death^6^ that is mediated by a number of proteases (called caspases) that cleave their target proteins at specific aspartate residues. In addition, autophagy-related proteins also interact with activated caspases that hinder or reinforce autophagy^7^. Autophagy can also increase the ability of tumour cells to overcome starvation and stress by preventing excessive protein degradation^8^. However, apoptosis or programmed cell death will occur in response to persistent activation of autophagy^9^. Nucleotides, amino acids and fatty acids, which are all the degradation products of autophagy, can be recycled via general cell metabolism^10^. Nucleotide biosynthesis and an acquired ability to synthesize fatty acids de novo are common cancer-related metabolic alterations that are important for rapidly proliferating cancer cells^11^. Amino acids regulate key metabolic pathways, and their metabolites (e.g., nitric oxide, polyamines and glutathione) are necessary for cancer cells to maintain growth, reproduction and immunity^12^. Importantly, intracellular levels of polyamines are maintained within very narrow limits, because decreases in polyamine levels interfere with cell growth, whereas excess levels of these compounds appear to be toxic^13,14^. In brief, the diverse changes in cellular metabolism affect tumour cell growth.

Fragile-site associated tumour suppressor (FATS) is a novel gene involved in cancer that is located at chromosome 10q26 and plays a key role in regulating tumour growth. The results of our previous study showed that FATS acts as a tumour suppressor involved in DNA-damage-induced tumourigenesis^15^, with FATS-mediated polyubiquitination of p53 promoting the activation of p53 in response to DNA damage^16^. Decreased FATS expression has been detected in breast cancer and was shown to be crucial for tumour growth, metastasis and therapy resistance. In our previous study, we demonstrated that low FATS expression is common in somatic NSCLC samples^17^. The low expression of FATS is associated with lymph node metastasis states and predicts the poor clinical outcome of NSCLC patients^17^, but little is known regarding the role of FATS in metabolic reprogramming in NSCLC. In the present study, we investigated how FATS affects cancer cell metabolism and elucidated how changes in polyamine metabolism inhibit NSCLC progression. We expect that the FATS-induced changes in polyamine metabolism can be exploited for cancer therapy.

## Methods

### Patients and tissue samples

In the present study, 154 NSCLC patients were recruited who underwent pulmonary surgery between 2005 and 2011 at the Tianjin Medical University Cancer Institute and Hospital. The study was approved by the Ethics Committee of the Tianjin Medical University, and written consent was obtained from all participants. All tissue samples used for analysis were obtained in the clinically indicated surgery and stored in liquid nitrogen until further use.

### Cell culture and transfection

Human NSCLC cell lines (A549, H520, H358, H460, H1299 and U87) were purchased from the American Type Culture Collection (ATCC, MD, USA). The cells were cultured in Dulbecco’s modified Eagle’s medium (DMEM, Invitrogen) supplemented with 10% foetal bovine serum (BI, ISR) and 1% penicillin/streptomycin (HyClone, Logan, Utah, USA) at 37 °C under an atmosphere with 5% CO_2_. A549 and H1299 cells were infected with a lentivirus expressing FATS that was purchased from Genomeditech (Shanghai, China). Cells were transfected with plasmids or small interfering RNA (siRNA; Hanbio, Shanghai, China) using Lipofectamine 3000 (Invitrogen, Carlsbad, CA, USA), according to the manufacturer’s instructions. The siRNA sequences used in this study are listed in Supplemental Table 1.

**Table 1:** Correlation of FATS mRNA expression and clinicopathological characteristics of lung cancer patients.

Variables	Low expression	High expression	*χ*^2^	*P*
*Sex*				
Female	29	20	0.104	0.747
Male	65	40		
*Age*				
<60	41	28	0.138	0.711
≥60	53	32		
*Smoking status*				
Never smoke	29	17	0.111	0.739
Smoke	65	43		
*Type of resection*				
Lobectomy	14	8	0.074	0.964
Pneumonectomy	77	50		
Bronchial sleeve resection	3	2		
*Lesion*				
Peripheral	29	17	0.111	0.739
Central	65	43		
*Histologic subtype*				
Squamous cell carcinoma	63	37	0.461	0.497
Adenocarcinoma	31	23		
*Location of tumour*				
Left	37	26	0.239	0.625
Right	57	34		
*Stage*				
I	23	17	5.240	0.073
II	26	25		
III	45	18		
*T stage*				
T1	30	11	4.006	0.135
T2	53	43		
T3	11	6		
*N stage*				
N0	40	27	7.919	0.019
N1	12	17		
N2	42	16		

### Plasmids and cloning

A Flag-FATS plasmid was constructed as previously described in detail^16^. A plasmid with an in-frame GFP-FATS fusion was constructed by inserting the full-length FATS cDNA into the vector pEGFP-C1 (Sigma, Buchs, Switzerland). A plasmid with an in-frame HA-ERβ fusion was constructed by inserting the full-length ERβ cDNA into the vector pCMV-HA (Addgene).

### RNA isolation and real-time reverse transcription PCR (RT-qPCR)

Total RNA extraction was performed using TRIzol Reagent (Invitrogen, San Diego, CA, USA). For reverse transcription, 5 μg of total RNA sample was reverse-transcribed into cDNA using M-MLV reverse transcriptase (ThermoFisher, Waltham, MA, USA)) according to the manufacturer’s protocol. Real-time PCR using SYBR Green I technology was then performed with the CFX96 Touch™ Real-Time PCR Detection System (BioRad). The mix was preheated at 95 °C (45 s) and amplified for 40 cycles at 95 °C (10 s) and 60 °C (40 s). The 2^−ΔΔCt^ method was used to calculate gene expression relative to the endogenous control. The PCR primers used in this study are listed in Supplemental Table 2.

**Table 2:** Overall survival and disease-free survival univariate analysis according to clinicopathologic factors in 154 lung cancer patients.

Variable	No. of patients	Percent (%)	6-Year OS rate (%)	*P* Value	6-Year DFS rate (%)	*P* Value
*Sex*						
Female	49	31.8%	24.5%	0.007	40.0%	0.003
Male	105	68.2%	42.9%		20.4%	
*Age*						
<60	69	44.8%	40.6%	0.398	37.7%	0.523
≥60	85	55.2%	34.1%		30.6%	
*Smoking status*						
Never smoke	46	29.9%	28.3%	0.090	26.1%	0.128
Smoke	108	70.1%	40.7%		37.0%	
*Type of resection*						
Pneumonectomy	22	14.3%	27.3%	0.067	27.3%	0.139
Lobectomy	127	82.5%	37.8%		33.9%	
Bronchial sleeve resection	5	3.2%	60.0%		60%	
*Lesion*						
Peripheral	108	70.1%	36.1&	0.584	33.3%	0.456
Central	46	29.9%	39.1%		34.8%	
*Histologic subtype*						
Squamous cell carcinoma	100	64.9%	38.0%	0.964	35.0%	0.965
Adenocarcinoma	54	35.1%	35.2%		31.5%	
*Location of tumour*						
Left	63	40.9%	34.9%	0.419	28.6%	0.214
Right	91	59.1%	38.5%		37.4%	
*Stage*						
I	40	26.0%	65.0%	<0.001	57.5%	<0.001
II	51	33.1%	45.1%		41.2%	
IIIA	63	40.9%	12.7%		12.7%	
*FATS expression*						
Low	94	61.0%	24.5%	0.001	21.3%	0.001
High	60	39.0%	56.7%		53.3%	

### Cell viability and proliferation assay

Cell viability was assessed with the CCK-8 assay (Cell Counting Kit-8, Hanbio, Shanghai, China). Cells were seeded in 96-well plates (1000 cells/well) and transfected with the control or FATS overexpression vector. CCK-8 was added for 4 h. The absorbance at 450 nm was measured for the samples collected on the indicated days using a Synergy H1 Hybrid Multi-Mode Microplate Reader (BioTek). All data were compared to the first OD measurement. Cells were plated in triplicate in 12-well plates at 1 × 10^4^ cells per well in 2 ml of medium. At the specific time points indicated in experiments, the wells were washed twice with PBS to remove dead cells, and then the entire contents of the well were trypsinized. Cell number was determined using a haemocytometer. For each well, the fold-change in cell number relative to Day 0 is reported.

### Cell apoptosis assay

Apoptotic cells were stained using a FITC Annexin V Apoptosis Detection Kit I (BD Pharmingen, San Jose, CA, USA) following the manufacturer’s instructions. Cell death was measured using a FACSVerse flow cytometer (BD Bioscience, San Diego, CA, USA) and quantified with FlowJo.

### Western blot assay

Western blotting was performed 72 h after transfection as specified. Cells were washed twice with ice-cold PBS and then lysed in RIPA buffer (20 mM Tris-HCl, pH 7.5, 150 mM NaCl, 1 mM EDTA and 1% Triton X-100), which contained a protease and phosphatase inhibitor cocktail (Thermo Scientific). Cleared supernatants were subjected to protein quantification using a BCA kit (Pierce). Equal amounts (50 µg) of total protein were fractionated by SDS-PAGE on an 8–12% gel, transferred to PVDF membranes (Roche, Basel, Switzerland), and then blocked in 5% non-fat dry milk/Tris-buffered saline plus Tween 20 (TBST). The membranes were incubated with the antibodies were incubated overnight at 4 °C. Where necessary, quantification was performed using ImageJ. The antibodies are described in Supplemental Table 3.

**Table 3:** Survival analysis of FATS.

	Overall survival		Disease-free survival	
Variables	HR (95% CI)	*P* Value	HR (95% CI)	*P* Value
Male	1.647 (1.081–2.509)	0.020	1.729 (1.142–2.619)	0.010
Stage	2.196 (1.654–2.916)	<0.001	2.105 (1.604–2.763)	<0.001
FATS expression	0.510 (0.323–0.805)	0.004	0.505 (0.324–0.787)	0.003

### Immunofluorescence assays

Measurements of autophagosome and autolysosome maturation were performed in cells transfected with the lentiviral reporter mRFP-GFP-LC3, which was purchased from HanBio Technology (Shanghai, China). In the green and red-merged images, autophagosomes are shown as yellow puncta (mRFP+/GFP+ spots), while autolysosomes are shown as red puncta (mRFP+/GFP− spots). In this assay, autophagic flux is increased when the number of both yellow and red puncta are increased in cells, while autophagic flux is blocked when only the number of yellow puncta is increased without red puncta alteration or when that of both yellow and red puncta is decreased in cells^18,19^.

Cells were plated in 96-well plates at 1000 cells per well. Subsequently, 16–18 h later, the cells were transfected with the control or FATS overexpression vector, and 48 h later were transfected with the mRFP-GFP-LC3. After 24 h, the cells were examined for the total number of autophagosomes (mRFP+/GFP+ spots) and autolysosomes (mRFP+/GFP− spots) compared to the total spots per cell (500 total spots from 5 cells per sample), with three independent experiments performed for each sample. DQ-BSA (red, D12051, Invitrogen, USA) experiments and quantification of intracellular proteolysis were performed as previously described^20^. Briefly, the pretreatment of cells was performed the same as that described for the LC3 reporter assay. Then, the cells were incubated with 10 µg/mL of DQ-BSA for 1 h at 37 °C, and nuclei were stained with DAPI (D9542, Sigma, St. Louis, MO, USA). All images were acquired on a PerkinElmer Operetta CLS microscope, and light microscopy was used for high-content imaging, with analysis performed using Harmony 4.5 (PerkinElmer, UK). Fluorescence intensity was normalized to the number of cell nuclei in each image, with a minimum of 100 cells analysed per sample. The total particle area per cell was determined from at least five fields that were randomly selected from different regions across the entirety of each sample using ImageJ. The RGB false-colour images were coloured with Image-Pro Plus 6.0.

### Amino acid replenishment assay

Amino acid (AA) replenishment was performed by incubating cells in normal DMEM (described as normal medium, NM), which contained a specific AA at a final concentration of 0.2 mM calculated relative to the levels present in DMEM (described as conditional medium, CM). Briefly, the cells were transfected with the control or FATS overexpression vector for 48 h, after which 20,000 cells in 250 µl of NM were seeded into the upper chamber of Transwell chambers with 8.0-μm PET membranes (353097, BD Falcon, USA), while CM was added to the lower chamber. After 6.5 h of incubation, cells on the top surface of the insert were removed by wiping with a cotton swab. The cells that had penetrated the bottom side of the membrane were fixed and stained using a 1% Crystal Violet Staining Solution (G1062, Solarbio, China). The numbers of penetrated cells were obtained using a Tissue Gnostics microscope (Zeiss) and calculated with ImageJ. The average of three random fields at ×100 magnification per membrane and the average of three membranes is presented.

### Immunoprecipitation assay

Transfected cells were washed in ice-cold PBS and scraped into RIPA lysis buffer supplemented with inhibitor cocktail (78430, Thermo, USA) on ice. Immunoprecipitation was performed using protein A/G agarose (Thermo) according to the manufacturer’s protocol. Briefly, 1000 µg of total protein in 500 µl of cell lysate was incubated overnight at 4 °C under agitation with magnetic beads conjugated with 1 µg of Flag, ODC, ERβ, or normal control IgG antibodies. After three washes with lysis buffer, the immunoprecipitated proteins were eluted with elution buffer provided by the manufacturer and processed for western blotting.

### Metabolomic analysis

Metabolomics assays were all performed as previously described^14,21^. Briefly, A549 cells were plated in 10-cm dishes after transfection with the control or FATS expression vector for 48 h. The Human Metabolome Technologies (HMT) metabolite extraction method for adherent cells was performed to extract metabolites. Metabolite concentrations were normalized to cell counts. For U^15^N_4_ tracings, U^15^N_4_-labelled medium containing 1 mM arginine (NLM-396, CIL, UK) was added. After 8 h of incubation, the cells were harvested to detect the abundances of pyrimidine nucleotides, amino acids and polyamine metabolites. The data were corrected for the natural abundance of the stable isotope, and the relative abundances of metabolites were compared.

### In vivo ubiquitination assay

A549 and H1299 cells expressing vector or FATS were transfected with His-tagged ubiquitin (His-Ub). Cell lysates were prepared 24 h after transfection and incubated with Ni-NTA beads (Qiagen, Venlo, Netherlands) in binding buffer (20 mM Tris-HCl [pH 7.4], 150 mM NaCl, 0.1% Triton X-100, 5 mM EDTA, 2 mM imidazole), which contained a protease and phosphatase inhibitor cocktail (78430, Thermo, USA). The beads were collected and washed four times with binding buffer. Then western blot assay was performed.

### Xenograft lung metastatic animal model

The experimental protocol used for nude mice was approved by the Ethics Committee of the Tianjin Medical University. All procedures involving animals and their care were conducted in accordance with the institutional guidelines that are in compliance with national and international laws and policies. Female BALB/c nude mice (5 weeks old) were purchased from the Tianjin Institute of Health and Environmental Medicine. A549 cells stably expressing GFP or GFP-FATS fluorescent protein in PBS were subcutaneously injected into the mice (7 mice per group) via the tail vein at a concentration of 2 × 10^6^ cells/mouse. After three weeks, tumorigenesis was observed in the mice in which the stable cell lines were implanted. Metastatic tumour size was measured every 2 days using a living animal imaging system after the formation of a tumour, and non-metastatic tumour size in mice was measured every 5 days after the tumour nodules could be observed. The tumour volume (mm^3^) of subcutaneous allografts was estimated according to a previously described formula^22^. At the final time point (12 weeks), the mice were sacrificed, and lungs were removed and fixed with 10% paraformaldehyde for further immunohistochemical analyses.

### Immunohistochemical analysis

For immunohistochemical staining, the tissue sections were dewaxed as previously described^23^. Subsequently, antigen retrieval was performed by boiling the slides in 10 mM sodium citrate (pH 6.0) at 130 °C for 3 min, after which the samples were pretreated with a 3% hydrogen peroxide solution for 30 min, rinsed, and then incubated with 5% normal goat serum for 20 min as a blocking agent. Then, the sections were incubated with a goat anti-ODC antibody (1:500; Santa Cruz Biotechnology) at 4 °C overnight, after which the slides were washed in PBS and incubated with the secondary antibody for 30 min at room temperature. All steps were preceded by rinsing of the sections with PBS (pH 7.4). The chromogen used in this assay was 3,3-diaminobenzidine (DAB). Haematoxylin was used as a counterstain, and after the dehydrated gum was sealed, the samples were observed and scored as previously described^24^ before being then imaged.

### Statistical analysis

All data were normalized to the control and are presented as the means ± SD. When comparing two groups to each other, Student’s *t*-test (unpaired, two-tailed) was performed. Survival curve statistical analysis was performed using the Kaplan–Meier test, with the *χ*^2^ test used to evaluate differences between the groups. The Cox proportional hazards ratio method was used to investigate the simultaneous effect of multiple predictors on survival. Differences where *P* < 0.05 were significant.

## Results

### FATS is associated with NSCLC progression and patient survival

**Fig. 1:**
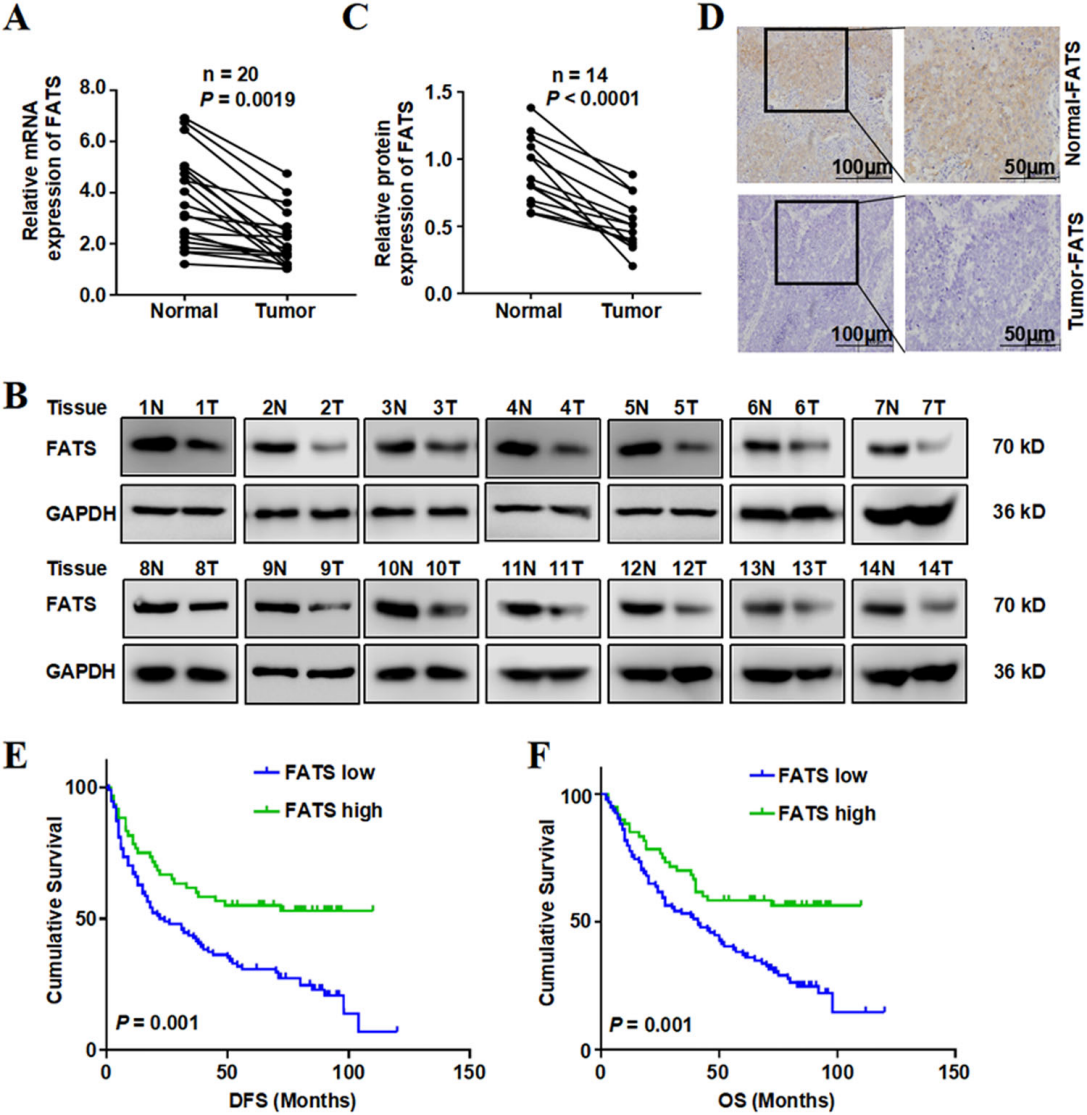
FATS is associated with NSCLC progression and patient survival. **a** RT-qPCR results of FATS mRNA levels in NSCLC patients and adjacent normal lung tissues (*n* = 20). **b**, **c** FATS protein levels in NSCLC patients and paired normal tissue samples (*n* = 14) analysed by western blotting. The graphs in (**c**) sow the quantification data. **d** Representative IHC micrographs from normal and tumour lung tissues. **e**, **f** Overall survival and disease-free survival were estimated using the Kaplan–Meier and the Cox proportional hazards ratio methods.

To investigate the role of FATS in NSCLC, we first assessed FATS expression at the mRNA level for 20 NSCLC samples and paired adjacent normal tissues through RT-qPCR analysis. FATS mRNA was significantly downregulated in NSCLC tissues compared with that observed in paired adjacent normal tissues (*P* = 0.0019; Fig. 1a). We further assessed FATS protein levels in 14 pairs of NSCLC tissues and paired adjacent normal tissues by western blot and observed that the changes in FATS protein levels were consistent with those observed for the mRNA levels (*P* < 0.0001; Fig. 1b, c). In addition, FATS was detected by IHC staining in 154 NSCLC samples. The results showed that 94 of 154 (61.0%) tumour samples had lower FATS level than that observed in adjacent normal lung tissues (Fig. 1d). The associations between FATS expression and the clinicopathological characteristics of NSCLC patients are summarized in Table 1. According to the retrospective analyses of the 154 NSCLC patients, we observed that high FATS expression was associated with the TNM stage (*P* = 0.019). NSCLC patients with high FATS expression had a lower tumour progression or longer overall survival time than those with low FATS expression (24.5% vs. 56.7%, 67 months vs. 41 months; *P* = 0.001), as revealed by the Kaplan–Meier analysis (Fig. 1e). The 6-year DFS rate and disease-free survival period of the high FATS group were significantly higher than those of the low FATS group (53.3% vs. 21.3%, 63.5 months vs. 23 months; *P* = 0.001; Fig. 1f, Tables 2 and 3).

### FATS inhibits NSCLC cell growth by promoting apoptosis

**Fig. 2:**
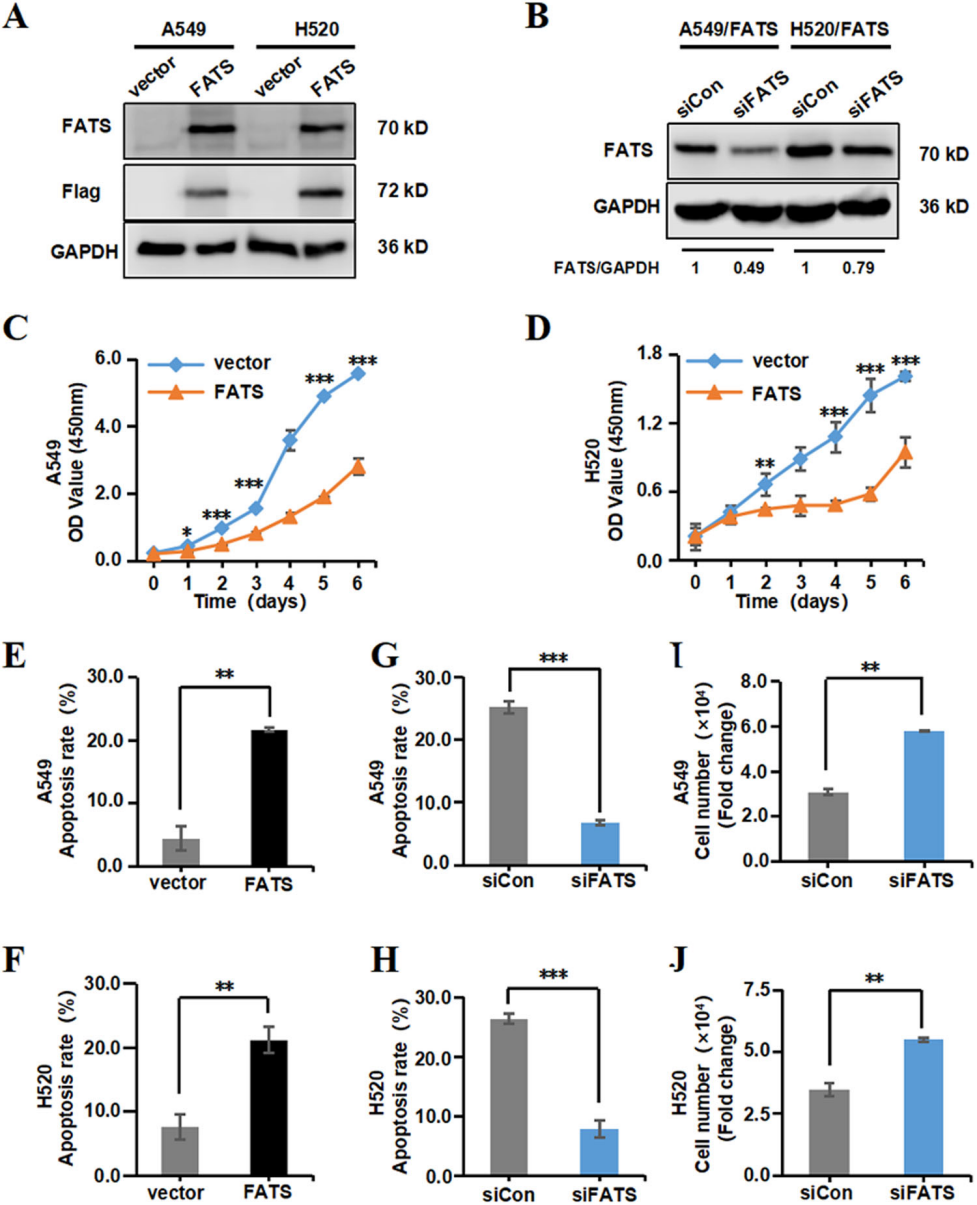
FATS inhibits NSCLC cell growth by promoting apoptosis. **a**, **b** Western blotting was performed to assess FATS expression in the indicated cell lines 72 h after transfection. GAPDH was used as a loading control. **c**, **d** The viability of A549 and H520 cells was assessed at the indicated timed using the Cell Counting Kit-8 assay after transfection. **e**–**h** Cell apoptosis was assessed by double staining with annexin V and propidium iodide (PI) followed by flow cytometry in which 10,000 labelled events were collected for the indicated cell lines 48 h after transfection. The percentage of apoptotic cells is shown, and the data are presented as the means ± SD of three independent experiments. **i**, **j** Proliferation of A549 and H520 cells transiently transfected with FATS siRNAs at the indicated time were determined by cell counting. All data are presented as the means ± SD of three independent experiments. *P < 0.05, **P < 0.01, ***P < 0.001; two-tailed unpaired Student’s *t*-test.

To characterize the specific contribution of FATS to tumour progression, FATS was overexpressed via transfection with a p3×Flag-FATS overexpression plasmid in A549, H520, H358 and H460 cells (Fig. 2a and S1A), and we selected A549 and H520 cells as the primary model for subsequent research. FATS-silenced models were also constructed in the indicated cells with FATS overexpression (Fig. 2b and S1B). To assess the influence of changes in FATS expression on the viability of NSCLC cells, cell viability was evaluated using the CCK-8 assay after transfection, and the data showed that when FATS was overexpressed, NSCLC cell viability was significantly decreased (Fig. 2c, d and S1C). We subsequently evaluated the potential role of FATS in cell apoptosis. Both NSCLC cell lines were transfected with the control and FATS overexpression vectors, and cell apoptosis was analysed by flow cytometry. As shown in Fig. 2e, f (Fig. S1D), the proportion of apoptotic cells transfected with the FATS overexpression vector was significantly higher than that observed in cells transfected with control vector. In addition, apoptotic cell death (Fig. 2g, h) and cell growth inhibition (Fig. 2i, j) induced by FATS could be partially blocked by transient depletion of FATS in A549 or H520 cells. These results suggested that FATS promotes cell apoptosis.

### FATS induces apoptosis via the pro-death autophagy pathway

**Fig. 3:**
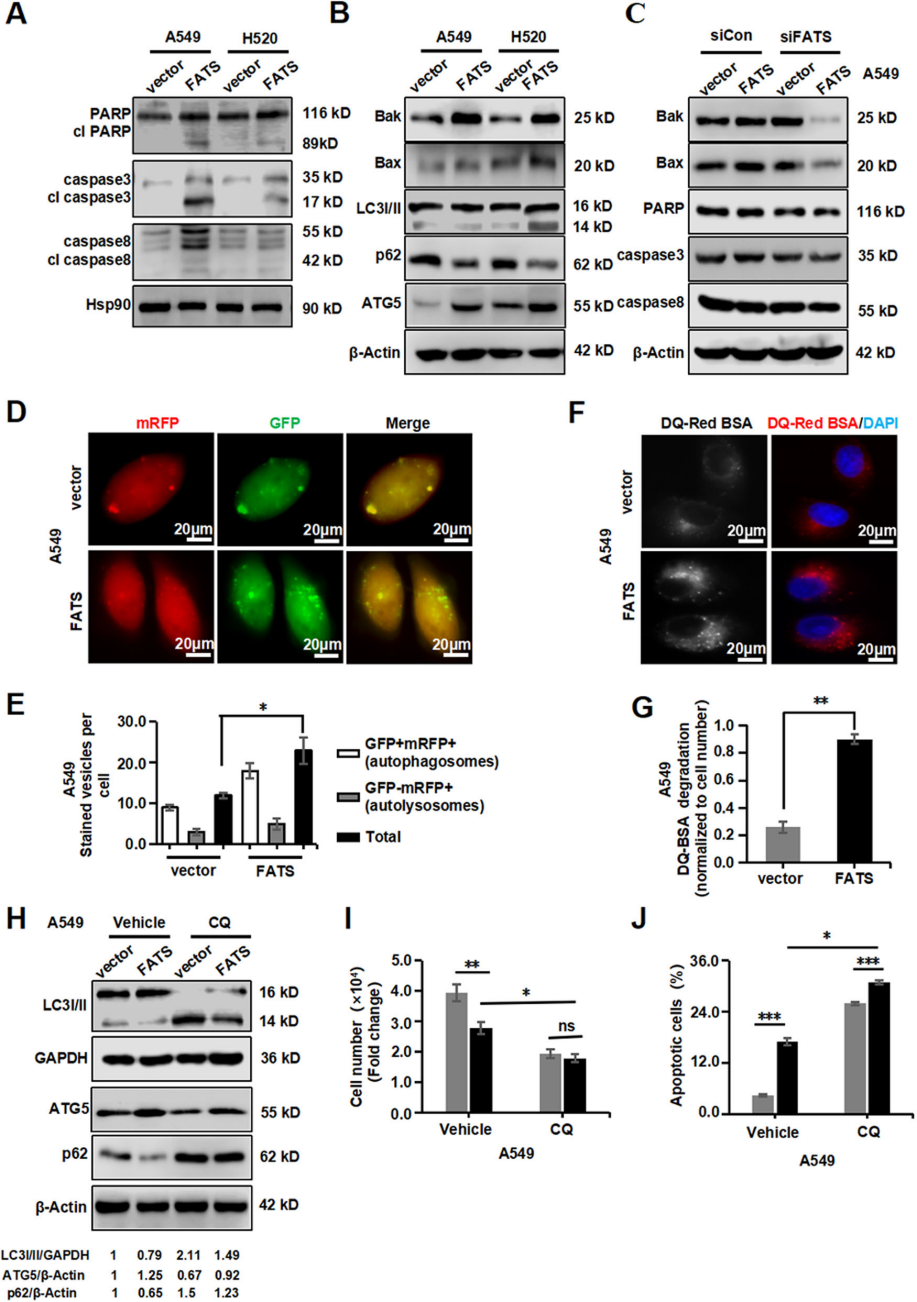
FATS induces apoptosis via the pro-death autophagy pathway. **a**–**c** Western blotting was performed to assess the apoptosis-related proteins in the indicated cell lines 72 h after transfection. Hsp90 and β-actin were used as loading controls. **d** The abundance of autophagosomes and autolysosomes was measured in A549 cells stably expressing an LC3-GFP-mRFP reporter and transfected with the control or FATS overexpression vector after 48 h. Autophagosomes are shown as yellow puncta (mRFP+/GFP+ spots), while autolysosomes are shown as red puncta (mRFP+/GFP− spots). Scale bar = 20 µm (*n* = 3). **e** Quantification of autophagosomes and autolysosomes in images that were obtained in (**c**) (*n* = 3). **f** DQ-BSA was used to measure intracellular autophagic flux in A549 cells 48 h after transfection. Cells were incubated with DQ-BSA and stained with DAPI. The RGB false-colour images were coloured with Image-Pro Plus 6.0. Scale bar = 20 µm (*n* = 3). **g** Fluorescence intensity was normalized to the number of cell nuclei in (**e**) (*n* = 3). **h** Western blotting was used to assess the levels of proteins in the autophagy-related signal pathway in A549 stable cell line expressing FATS and treated with or without CQ (10 µM). GAPDH was used as a loading control for LC3I/II, while β-actin was used as a loading control for p62 and ATG5. **i** Cell counting was performed to assess the influence of CQ on A549 cells transiently transfected with FATS siRNAs. **j** CQ could rescue FATS-induced cell apoptosis, as measured by annexin V/PI staining and flow cytometry. All data are presented as means ± SD. *P < 0.05, **P < 0.01, ***P < 0.005; two-tailed unpaired Student’s *t*-test.

To elucidate the mechanism for the pro-apoptotic effect of FATS on NSCLC cells, we first examined the effects of FATS on the expression levels of the caspase and Bcl-2 family proteins, some of which exhibit pro-survival functions while others exhibit pro-apoptotic functions^25,26^. The results showed that cleaved caspase-8/-3 and PARP, all of which are markers of apoptosis activation, were detected upon FATS overexpression, and significant changes in pro-caspase-8/-3 and pro-PARP indicated the occurrence of apoptosis (Fig. 3a). Correspondingly, the levels of the pro-death Bcl-2 proteins Bax and Bak were increased significantly (Fig. 3b). The above findings indicated that FATS expression may induce apoptosis in NSCLC cells.

Autophagy is considered to be another form of programmed cell death^27,28^ that is involved in a specific mode of death called autophagic cell death^29,30^. We hypothesized that autophagy is also involved in FATS-induced apoptosis in NSCLC. To test this hypothesis, we first examined the autophagy markers LC3I/II and ATG5 which were all exhibited elevated levels, and accordingly, p62 protein levels were decreased when FATS was enhanced (Fig. 3b). This autophagy-promoting activity was abrogated by FATS knockdown (Fig. 3c). Moreover, the level of autophagy was measured using a mRFP-GFP-LC3 reporter to detect the accumulation of mature autophagic vesicles (Fig. 3d and S2A). The results showed that FATS overexpression increased the formation of LC3-positive mature autophagosomes and autolysosomes (Fig. 3e and S2B).

We analysed autophagic flux in the presence and absence of FATS or the autophagy inhibitor chloroquine (CQ) to distinguish between the induction of autophagy and the blockage of degradation. The results showed that FATS significantly increased autophagic flux, which was demonstrated by increased DQ-BSA dequenching (Fig. 3f, g and S2C, D). We also noted that LC3-II accumulation was promoted by CQ treatment compared to that observed by FATS overexpression alone (Fig. 3h), suggesting that FATS indeed induced autophagy rather than blocked degradation. Furthermore, CQ significantly attenuated the inhibition of cell growth caused by FATS overexpression (Fig. 3i) and the influence of FATS on apoptosis (Fig. 3j). Together, these data suggested that FATS can promote apoptotic cell death via the pro-death autophagy pathway.

### FATS regulates polyamine metabolism in an ODC-mediated manner

**Fig. 4:**
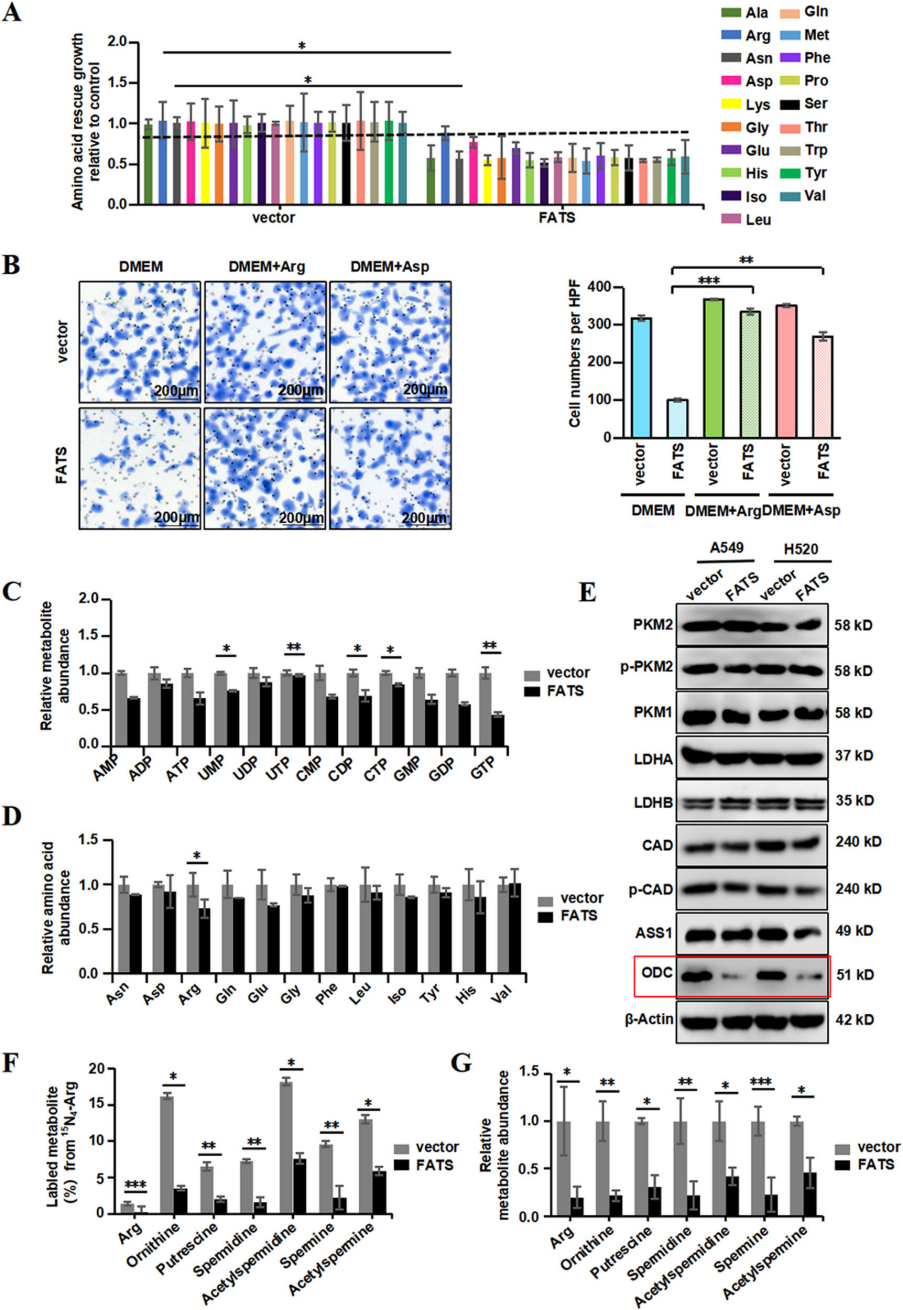
FATS regulates polyamine metabolism in an ODC-regulated manner. **a** Proliferation of A549 cells overexpressing FATS relative to with the vector control grown in the conditional medium containing additional specific amino acids (19 in total) separately at a final concentration of 0.2 mM for 48 h. The results are the average of three biological replicates. **b** Amino acid requirement of arginine or aspartate was assessed using Transwell Chambers assay after transfection with the control or FATS overexpression vector for 48 h in A549 cells. Scale bar = 200 µm (*n* = 3). **c**, **d** Mass isotopomer analysis of U^15^N_4_-L-arginine scintillation counts in A549 cells expressing the control or FATS overexpression vector and cultured in medium containing 1 mM labelled arginine at 10 h (normalized to cell number). The results are the average of three biological replicates. **e** Western blots of A549 and H520 cell lines with the control or FATS overexpression vector and cultured in medium containing 1 mM arginine for 72 h. **f**, **g** Mass isotopomer analysis of U^15^N_4_-L-arginine scintillation counts in A549 cells with the control or FATS overexpression vector and cultured in medium containing 1 mM labelled arginine at 10 h (normalized to cell number). The results are the average of three biological replicates. All data are presented as means ± SD. *P < 0.05, **P < 0.01, ***P < 0.005; two-tailed unpaired Student’s *t*-test.

After demonstrating that autophagy cannot sustain cell survival during FATS overexpression, we next examined a series of metabolites to identify which contribute to restoring cell growth. Of all the metabolites analysed, treatment of A549 and H520 cells with amino acids indicated that arginine and aspartate met our expectations (Fig. 4a and S3A). In addition, to assess the uptake of arginine or aspartate, another individual amino acid assay was performed using the Transwell filter system without Matrigel. The results demonstrated that arginine and aspartate uptake by FATS-overexpressing cells was comparable to that of the control cells, indicating that arginine and aspartate had a notable effect (Fig. 4b and S3B).

We hypothesized that FATS inhibited NSCLC growth by disrupting the supply of nitrogen, which is required for nucleotide biosynthesis-associated cell proliferation. To test this hypothesis, we performed metabolite tracing experiments in medium containing nitrogen-15-labelled (U^15^N_4_) arginine to gain insight into the metabolic fate of nitrogen. Analysis of metabolites derived from arginine catabolism displayed fewer labelled nucleotides relative to vector-only controls (Fig. 4c). In accordance with decreased arginine uptake in FATS-overexpressing cells, the cells also displayed decreased labelled amino acid abundance compared to the vector-only controls (Fig. 4d). Taken together with the above results, we deduced that FATS may cause changes in the metabolic link between the urea cycle and cancer cell proliferation by reducing aspartate availability for pyrimidine synthesis^31^. To test this possibility, we next assessed the protein levels of enzymes related to aspartate-mediated pyrimidine synthesis. However, the results were surprising due to inconspicuous protein levels, such as those observed for the urea cycle rate-limiting enzyme ASS1, CAD (aspartate transcarbamylase, carbamoyl-phosphate synthase 2), phosphate-CAD, etc., except for ODC (ornithine decarboxylase), which is crucial for polyamine catabolism and displayed a significant decrease when FATS was overexpressed (Fig. 4e). Furthermore, citrulline, argininosuccinate and fumarate, which are all involved in the urea cycle, displayed slight changes in relative metabolite abundance (Fig. S4A). These findings suggest that FATS expression had a more remarkable influence on the arginine metabolic pathway than the urea cycle.

Considering that the cells with high expression of FATS will gradually die during the process of cell culture, leaving the cells which were insufficient expression of FATS, to assess the metabolic changes of polyamines induced by FATS, we constructed A549 cells that stably overexpressed FATS (Fig. S4B) and set up ^15^N-labelling metabolic analysis to trace the fate of polyamine related metabolites. As expected, our results showed that the labelled metabolite levels of putrescine, spermidine, spermine, acetylspermidine and acetylspermine, which are all derived from Arg, were decreased to different degrees in the FATS overexpression group compared with the vector control group (Fig. 4f). The relative abundances of the abovementioned metabolites were also reduced (Fig. 4g). Collectively, these data show that FATS regulates polyamine metabolism in an ODC-mediated manner.

### FATS, ERβ and ODC bind to each other

**Fig. 5:**
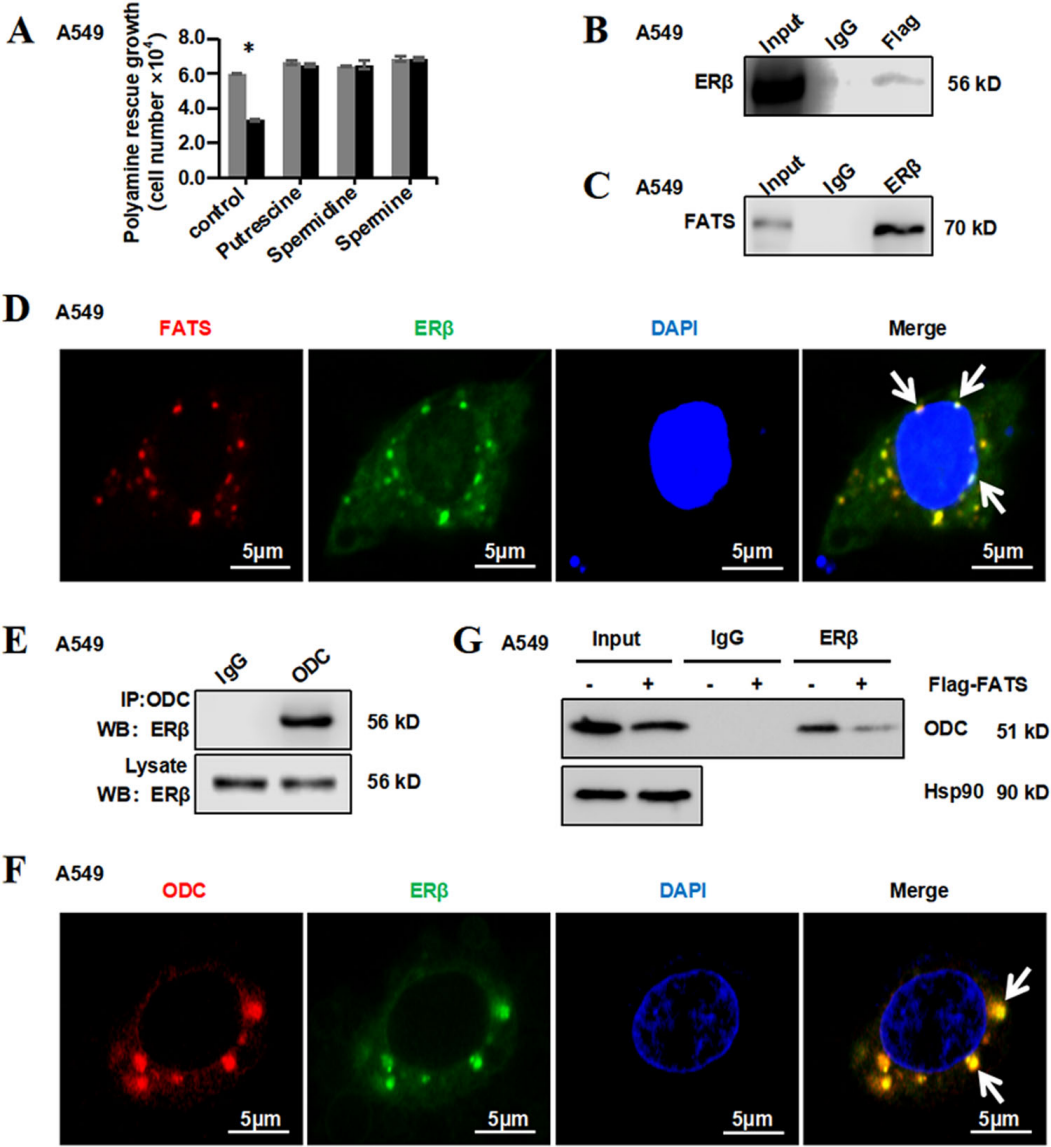
FATS, ERβ and ODC bind to each other. **a** Proliferation of A549 cells with the control or FATS overexpression vector and cultured in medium containing the indicated polyamine for 72 h. **b**, **c** A549 cells were transfected with the control or Flag-FATS vector for 48 h. Then, cell lysates immunoprecipitated against Flag or ERβ-specific antibodies, followed by immunoblotting using antibodies against ERβ or FATS (*n* = 3). **d** A549 cells overexpressing FATS were subjected to immunofluorescence analysis. Cells were stained with antibodies against ERβ and FATS, and nuclei were stained with DAPI (scale bar, 5 µm; *n* = 3). **e** Co-immunoprecipitation of ODC with ERβ in A549 cells after transfection with the control or Flag-FATS vector after 48 h (*n* = 3). **f** A549 cells expressing FATS were transfected with an HA-ERβ vector for 48 h, after which the co-transfected cells were subjected to immunofluorescence analysis. Cells were stained with antibodies against ERβ and ODC, and nuclei were stained with DAPI (scale bar, 5 µm; *n* = 3). **g** A549 cells with the control or FATS overexpression vector were subjected to immunoprecipitation analysis. Cells were incubated with an ERβ-specific antibody, which was followed by immunoblotting using an antibody against ODC. Hsp90 was used as loading control (*n* = 3).

Based on the effects of metabolic rewiring of polyamine (Fig. S1A) on cell survival, subsequent assays were performed in which the addition of putrescine (5 mM), spermidine (2 mM) or spermine (2 mM), which are three major polyamines in the culture medium, were shown to rescue the inhibition of cell growth induced by FATS overexpression (Fig. 5a). Our results also showed the restorative trend of metabolites related to pyrimidine nucleotides (Fig. S5B) and amino acid abundances (Fig. S5C) with the addition of these three major polyamines. These results further demonstrated that FATS plays an important role in the regulation of polyamine metabolism.

Having demonstrated that FATS regulates the expression of ODC, we subsequently performed immunoprecipitation assays to assess whether FATS directly binds to ODC, and the result was negative (data not shown). FATS is primarily localized in the nucleus^16^, whereas ODC is localized in the cytoplasm and membrane^32^. Therefore, we next investigated how FATS can indirectly regulate ODC.

Based on the findings that nuclear and membrane receptor-mediated signalling pathways modulate polyamine biosynthesis and interconversion^33^, we first tested whether FATS interacts with three nuclear receptors, androgen receptor (AR)^34^, oestrogen receptor (ER, two isoforms, alpha and beta)^35^ and Glu-cocorticoid receptor (GR)^36^, which are reported members of the nuclear receptor family. Consistent with our hypothesis, FATS was confirmed to interact with ERβ in NSCLC cells (Fig. 5b, c), while AR and GR interactions were not detected (data not shown). Moreover, the FATS–ERβ complex was localized in the perinuclear space (Fig. 5d), providing the possibility for it to regulate ODC, and the immunoprecipitation assay demonstrated ODC binding to ERβ (Fig. 5e). The subcellular localization of ODC and ERβ was assessed by immunofluorescence assays, the colocalization of these proteins in the perinuclear space could be clearly observed from the image (Fig. 5f). Subsequently, co-immunoprecipitation analysis was performed and revealed that FATS, ERβ and ODC bind to each other (Fig. 5g). Taken together, these results demonstrate the interaction of the FATS, ERβ and ODC proteins in NSCLC cells.

### FATS regulates ODC in an ERβ-dependent manner

To fully elucidate the molecular determinants responsible for the regulation of ODC by FATS, we assessed ODC expression at both the mRNA and protein levels. ODC is regulated by p53 at the mRNA level^37^, and p53 was shown to be regulated by FATS via ubiquitin signalling in our previous study^16^. Thus, we assessed whether the inhibition of ODC depends on p53. Two NSCLC cell lines, A549 and H1299 (p53+ and p53-null, respectively) stably overexpressing FATS in the following experiments. The result of FATS overexpression in the A549 and H1299 cells are shown in Fig. S4B.

**Fig. 6:**
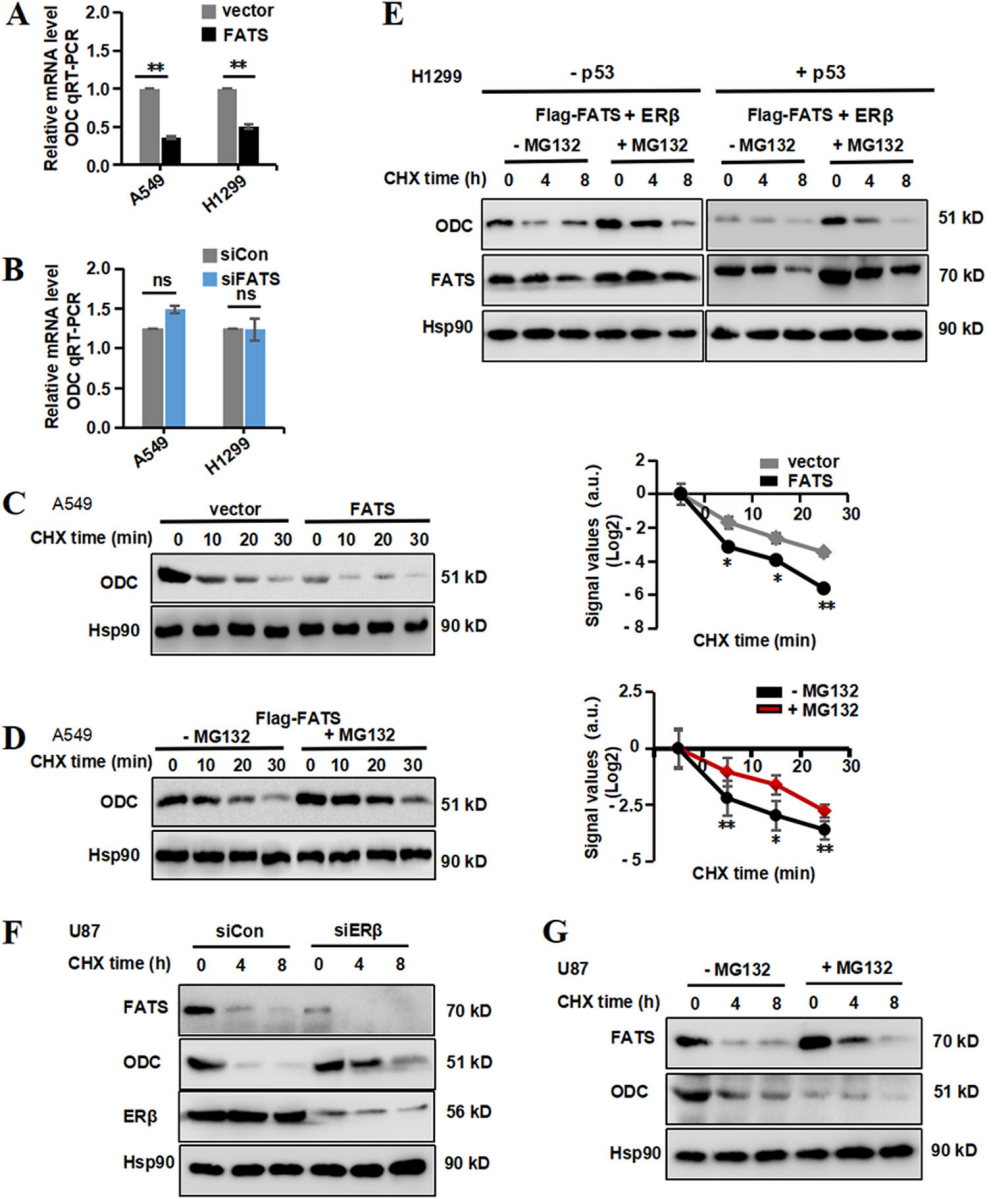
FATS regulates ODC in an ERβ-dependent manner. **a** The ODC mRNA levels in A549 and H1299 NSCLC cells stably transfected with the control or FATS overexpression vector were analysed by quantitative reverse transcription PCR (RT-qPCR) and normalized to GAPDH expression. **b** A549 and H1299 cells stably transfected with the FATS overexpression vector were transfected with control or FATS siRNA for 48 h and then analysed by RT-qPCR to assess ODC mRNA levels. **c**, **d** Representative western blot showing the stability of ODC in A549 cells stably transfected with the control or FATS overexpression vector and cultured in medium containing CHX (5 µg/mL) or MG132 (20 μM) at the indicated time (*n* = 3). Quantitative analysis of the western blot results was performed using ImageJ and relative densitometric quantification of ODC protein levels is shown in the right panel. **e** H1299 cells stably transfected with the control or FATS overexpression vector alone or in combination with either p53 or ERβ constructs. The vectors were co-transfected to avoid transfection artefacts. After 48 h, the cells were treated with CHX (5 µg/mL) or MG132 (20 μM) at the indicated time. The protein levels of ODC, FATS and Hsp90 were analysed by western blot, with Hsp90 used as a loading control. **f** U87 cells were transfected with control siRNA or ERβ siRNA as indicated for 48 h and then treated with CHX (5 µg/mL) at the indicated time. The whole-cell lysates were harvested, and the protein levels of ODC, FATS and ERβ were analysed by western blot, with Hsp90 used as a loading control. **g** U87 cells were treated with CHX (5 µg/mL) or MG132 (20 μM) at the indicated time. The protein levels of ODC, FATS and Hsp90 were analysed by western blot, with Hsp90 used as a loading control. All data are presented as means ± SD. *P < 0.05, **P < 0.01, ***P < 0.005; two-tailed unpaired Student’s *t*-test.

We first evaluated the effect of FATS on ODC mRNA levels, and the results showed that FATS significantly downregulated ODC mRNA in the p53+ and p53− cell lines (Fig. 6a). We repeated the experiment using a siRNA targeting FATS and observed that siFATS did not decrease ODC mRNA levels in either cell line (Fig. 6b). Subsequently, we assessed ODC protein instability in A549 cells. In the presence of cycloheximide (CHX), an inhibitor of protein synthesis, FATS overexpression dramatically decreased the basal ODC protein stability (Fig. 6c), and a reduced half-life can also be observed in the right panel, demonstrating that FATS destabilizes ODC protein. We also showed that the FATS inhibition-dependent decrease in ODC protein levels was rescued by treatment with the proteasome inhibitor MG132 (Fig. 6d). Because the A549 cell line is both p53+ and ERβ+, H1299 cells stably overexpressing FATS that were co-transfected with ERβ or p53 constructs were used to determine whether FATS also controls ODC degradation in p53-null cancer cells and to elucidate the role of ERβ, which is involved in suppressing ODC levels. Our results showed that FATS could accelerate ODC degradation in a p53-independent manner, and ODC levels decreased more notably in the presence of ERβ. The addition of MG132 could also rescue the decreased protein level of ODC (Fig. 6e). Furthermore, FATS also decreased ODC protein levels in U87 cells, which are wild type for FATS, and the FATS-mediated suppression of ODC was eliminated when ERβ was silenced (Fig. 6f), and MG132 treatment could also rescue the suppression of ODC (Fig. 6g). These results indicate that FATS can decrease the stability of ODC protein in an ERβ- but not p53-dependent manner.

The ubiquitin-independent nature of ODC degradation by the proteasome is well-known^38^. The degradation of ODC is likely to be accelerated in the presence of FATS which was shown in Fig. 6c. Moreover, FATS is an E2-independent ubiquitin ligase^16^. To determine whether ODC is ubiquitinylated by FATS and ODC degradation is ubiquitin-dependent, we have performed vivo ubiquitination assay in A549 and H1299 cells which expressing vector or FATS were transfected with His-tagged ubiquitin (His-Ub). Our results showed that FATS-mediated degradation of ODC was independent of polyubiquitination, even with the treatment of MG132 (Fig. S6A,B), indicating that FATS mediated the degradation of ODC protein is not in the ubiquitin-dependent pathway. Antizyme is a polyamine-induced cellular protein that binds to ODC, and targets it to rapid ubiquitin-independent degradation by the 26S proteasome, in which especially antizyme 1 (AZ1) plays a major role^39^. We next examined the effects of AZ1 on the stability of ODC which is medicated by FATS and ERβ in U87 cells by knocking down of AZ1 using a small interfering RNA. The degradation of ODC was suppressed when AZ1 was silenced (Fig. S6C). Knockdown of AZ1 in A549 and H1299 with the presence of p53 and ERβ cells also blocked the degradation of ODC (Fig. S6D,E). These results demonstrated that the degradation of ODC was accelerated by AZ1, as reported previously^40^. Taken together, our results indicate that FATS–ERβ complex accelerates ODC degradation by AZ1.

### FATS suppresses NSCLC tumour growth and metastasis in vivo

**Fig. 7:**
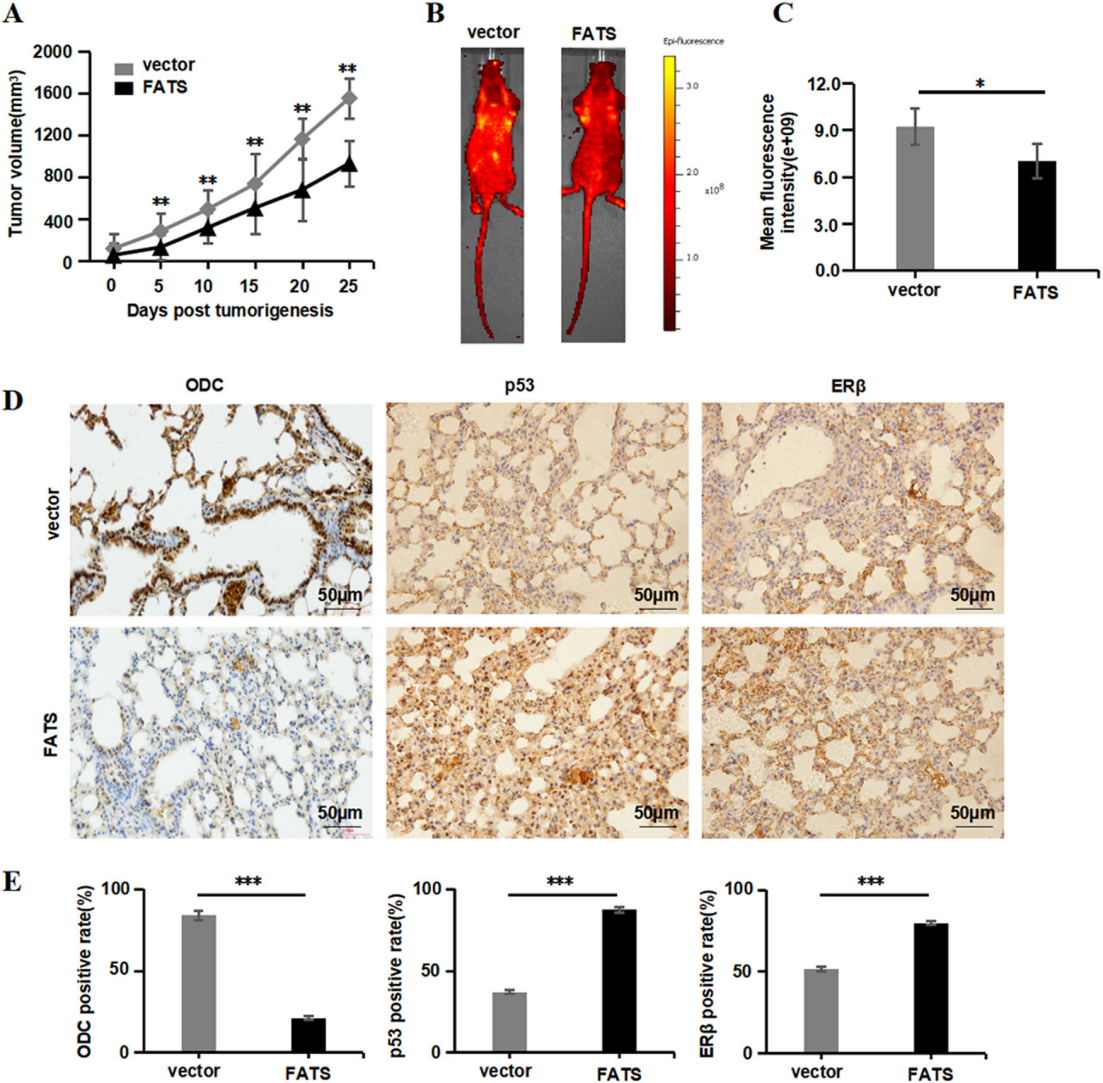
FATS suppresses NSCLC tumour growth and metastasis in vivo. **a** Estimated tumour volumes (mm^3^) of the subcutaneous allografts of A549 cells stably expressing GFP or GFP-FATS in BALB/c nude mice. *n* = 7 per group. **b** The sizes of metastatic tumours size resulting from tail vein injection of A549 cells expressing GFP or GFP-FATS were measured using a living animal imaging system at the indicated time. Mice with intrapulmonary metastases were anaesthetized and imaged using the Xenogen IVIS Spectrum imaging system. *n* = 7 per group. **c** Quantification of fluorescence signal intensity for the two groups. **d** Immunohistochemical analysis of ODC in the FATS and matched vector groups at the end of the experiment shown in (**b**) (*n* = 3; scale bar, 50 µm). **e** IHC staining results were evaluated as the percentage of positive cells. Scale bar, 50 µm. All data are presented as means ± SD. *P < 0.05, **P < 0.01, ***P < 0.005; two-tailed unpaired Student’s *t*-test.

To evaluate the role of FATS in tumour growth and metastasis in vivo, FATS was overexpressed in NSCLC xenograft models. Consistent with our in vitro observations, the tumour xenograft models revealed that FATS suppressed the tumorigenicity of A549 cells in nude mice. Xenografts from mice injected with FATS-overexpressing cells were much smaller than those injected with the vector control cells (Fig. 7a). A lung metastasis model by tail vein injection was constructed to assess the effect of FATS on the metastatic potential of A549 cells. The metastases, which were observed by yellow fluorescence in the lungs, indicated that the intrapulmonary metastasis in the FATS overexpression group was less than that observed in the vector control group (Fig. 7b, c), demonstrating that FATS inhibits NSCLC tumour growth and metastatic properties in vitro. In addition, we also performed an immunohistochemical assay to analyse the expression of ODC, p53 and ERβ in the implanted tumour cells expressing GFP or GFP-FATS. Consistent with our previous hypothesis, we observed that ODC expression the FATS overexpression group was reduced compared to that observed in the control group, and high p53 protein levels were notably correlated with high FATS expression (Fig. 7d, e). Taken together, these data indicated that ODC expression was inhibited by FATS overexpression, which suppressed cell growth and metastasis in human NSCLC.

**Fig. 8:**
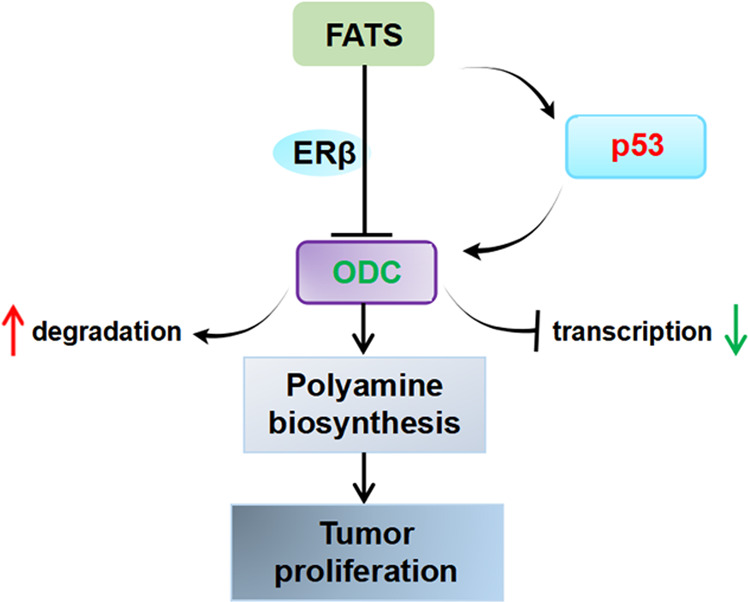
Model of FATS-induced polyamine metabolic changes in NSCLC. FATS regulates polyamine metabolism in two ways: (i) FATS promotes the degradation of ODC protein in an ERβ-dependent manner; (ii) FATS-mediated polyubiquitination of p53 promotes the activation of p53 and inhibits the transcription of ODC. Downregulation of ODC leads to the decreases synthesis of polyamine from arginine and inhibits cancer cell proliferation.

In summary, our results showed that FATS acts as a tumour suppressor through the suppression of polyamine biosynthesis by inhibiting ODC at the protein and mRNA levels, which is dependent on ERβ but partially independent of p53 (Fig. 8).

## Discussion

Tumour proliferation can be disrupted by apoptosis or nutrient limitation^41^. Although the functions of FATS in regulating p53 in ubiquitin signalling were reported in our previous study^16^, the roles of this oncogene in apoptosis or metabolism have remained unknown. To explore the role of FATS in these complex processes, we evaluated the expression of a number of genes associated with apoptosis and autophagy. Our results showed that FATS promotes apoptosis and enhances autophagy, which corresponded to a significant inhibition of cell proliferation.

To elucidate how FATS inhibits lung cancer cell proliferation and to identify the component that it interacts with to promote this inhibition, we performed amino acid addition and LC-MS assays and demonstrated that arginine is a clear nutrient requirement for FATS overexpression in cells. The ability of lung cancer cells to convert arginine to glucose provides a metabolic advantage by generating substrate for glycolysis. Surprisingly, the western blotting results revealed no observable changes in metabolic enzyme genes related to cell proliferation, including those involved in the tricarboxylic acid cycle and aspartate biosynthesis^42^.

As previously reported, L-arginine is a versatile amino acid that serves as a building block for protein synthesis and is a precursor for multiple metabolites, including polyamines and nitric oxide (NO), which have strong immunomodulatory properties^43,44^. Further LC-MS analyses demonstrated that FATS inhibits polyamine metabolism, which corresponded to downregulated level of ODC protein, which is the first rate-limiting enzyme of polyamine metabolism^21,45^. The IP assay results revealed a novel association between FATS and ODC in NSCLC. Notably, FATS activity was shown to be dependent on ERβ, a nuclear receptor used for protein translocation into the cytoplasm, and resulted in decreased ODC expression.

To further evaluate the role of FATS in tumorigenesis and metastases in vivo, FATS was overexpressed in A549 xenograft models. FATS overexpression inhibited tumour growth, which was consistent with a low fluorescence density and indicated fewer metastases in the lung. The ODC protein level detected in lung solid tumours was lower than that observed in the normal adjacent tissue, indicating that the availability of FATS for tumour growth is rate-limiting. These results demonstrate that FATS overexpression inhibits ODC in solid tumours, findings that corresponded with our in vitro results.

Taken together, these results support a model of polyamine metabolic changes upon FATS expression in which FATS degrades ODC protein in an ERβ-dependent manner, while FATS-mediated polyubiquitination of p53 promotes its activation and inhibits ODC transcription. The downregulation of ODC depresses the synthesis of polyamines derived from arginine and downregulates the metabolism of nitrogen in purine and pyrimidine synthesis, which are all precursors used in aspartate biosynthesis that are involved in cell proliferation. These results demonstrated that FATS mediates ODC suppression in NSCLC and suggests that modulation of FATS–ODC-mediated polyamine metabolism could be a therapeutic strategy for NSCLC. And further research is needed to clarify how FATS–ERβ–ODC complex associates with AZ1 for proteasomal ODC degradation and to explore its potential applications in cancer treatment.

In summary, the results of our study and others demonstrated that intracellular levels of polyamine are essential for sustaining cell growth. While these levels were lower in cells with FATS overexpression than in control cells, the balance of nutrients was disturbed. FATS acts as a suppressor of polyamine biosynthesis, leading to enhanced apoptosis and the suppression tumorigenesis. Further studies are needed to elucidate the specific domains of FATS associated with ODC regulation and to understand how the pharmacological inhibition of this pathway sensitizes NSCLC tumours to FATS therapy, which represents a potential therapeutic approach for NSCLC patients.

## Supplementary information

Figure S1–6 Legends

revise-Figure-S01

revise-Figure-S02

revise-Figure-S03

revise-Figure-S04

revise-Figure-S05

revise-Figure-S06

siRNA

antibody list

RT-qPCR Primers
