# Supplementary material for: FATS regulates polyamine biosynthesis by promoting ODC degradation in an ERβ-dependent manner in non-small-cell lung cancer
Source: Cell Death Dis. 2020 Oct 9;11(10):839. doi: 10.1038/s41419-020-03052-1 (PMC7547721; doi:10.1038/s41419-020-03052-1)
Supplement: Supplementary file 1 — Figure S1–6 Legends [file 41419_2020_3052_MOESM1_ESM.doc]

**Figure Supplement Legends**

**Figure S1 FATS inhibits NSCLC cell growth by promoting apoptosis**

(A) and (B) Western blotting was used to detect the expression of FATS in H358 and H460 cell lines 72 h after transfection. GAPDH was used as a loading control.

(C) Cell viability was detected in H358 and H460 cells at the indicated time by the Cell Counting Kit-8 Assay after transfection. Data are the mean ± SD of three independent experiments. *p < 0.05, **p < 0.01, ***p < 0.001; two-tailed unpaired Student’s *t*-test.

(D) Cell apoptosis was detected by double staining with annexin-V and propidium iodide (PI) and subjected to flow cytometric analysis that collected 10,000 events labeled in indicated cell lines 48 h after transfection. The percentage of apoptotic cells is shown. Data are the mean ± SD of three independent experiments.

**Figure S2 FATS induces apoptosis via the pro-death autophagy pathway**

(A) and (B) The abundance of autophagosomes and autolysosomes was measured with that stably expressed an LC3-GFP-mRFP reporter in 520 cells transfected with vector or FATS after 48 h and quantification of images obtained in (A) (n = 3).

(C) and (D) Intracellular autophagy flux in H520 cells tested using DQ Red BSA and quantification of images obtained in (D) (n = 3).

All data represent means ± SD. **P* < 0.05, ***P* < 0.01, ****P* < 0.005; two-tailed unpaired Student’s *t*-test.

**Figure S3 FATS regulates polyamine metabolism in an ODC-regulated manner**

(A) Proliferation of H520 cells overexpressing FATS relative to with the vector control grown in the conditional medium containing additional specific amino acids (19 in total) separately at a final concentrations of 0.2 mM for 48 h. The results are the average of three biological replicates.

(B) Amino acid requirement of arginine or aspartate was assessed using Transwell Chambers assay after transfection with the control or FATS overexpression vector for 48 h in H520 cells. Scale bar = 200 µm (n = 3).

**Figure S4 FATS cause no changes in arginine metabolism**

1. Arginine metabolite was traced using mass isotopomer analysis of U^15^N_4_-L-Arginine scintillation count in A549 cells expressing vector or FATS cultured in medium containing 1 mM labelled-arginine after 10 h (normalized to cell number). Results are the average of three biological replicates.
2. A549 and H1299 cells were infected with lentivirus of vector or FATS for 48 h, then the cells were cultured with the medium containing puromycin (1 µg/mL) for 7 days. The whole cell lysate were harvested and the protein levels of Flag and FATS were analysed by western blot. GAPDH was used as loading control.

**Figure S5 FATS cause no changes in polyamines metabolites related to pyrimidine nucleotides and amino acid abundances**

1. A model of polyamine metabolic.
2. and (C) Mass isotopomer analysis of U^15^N_4_-L-Arginine scintillation count in A549 cells expressing vector or FATS cultured with the medium containing 1 mM of labelled-arginine at 10 h (normalized to cell number). Polyamines metabolites related to pyrimidine nucleotides and amino acid abundances were analyzed. Results are the average of three biological replicates.

**Figure S6 AZ1 could accelerate the degradation of ODC which is regulated by FATS**

(A) and (B) A549 and H1299 cells expressing vector or FATS were transfected with His-tagged ubiquitin (His-Ub), respectively. After 24 h, cells were either untreated or treated with MG132 (20 mM) for 6 h. His-tagged and ubiquitin-conjugated proteins were purified by Ni-NTA beads, followed by immunoblotting with an ODC antibody. The levels of indicated proteins in cell lysates are shown in the lower panel. (C) U87 cells were transiently transfected with AZ1 siRNA. After 48 h, cells were treated with CHX (5 µg/mL) at the indicated time. The protein levels of ODC, AZ1 and Hsp90 were analysed by western blot, with Hsp90 used as a loading control. (D) A549 cells expressing vector or FATS were transiently transfected with AZ1 siRNA. After 48 h, cells were treated with CHX (5 µg/mL) at the indicated time. The protein levels of ODC, AZ1 and Hsp90 were analysed by western blot, with Hsp90 used as a loading control. (E) H1299 cells expressing FATS/ERβ/p53 were transiently transfected with AZ1 siRNA. After 48 h, cells were treated with CHX (5 µg/mL) at the indicated time. The protein levels of ODC, AZ1 and Hsp90 were analysed by western blot, with Hsp90 used as a loading control.All data are presented as the means ± SD. **P* < 0.05, ***P* < 0.01, ****P* < 0.005; two-tailed unpaired Student’s *t*-test.
