# Supplementary figures and images for: FATS regulates polyamine biosynthesis by promoting ODC degradation in an ERβ-dependent manner in non-small-cell lung cancer
Source: Cell Death Dis. 2020 Oct 9;11(10):839. doi: 10.1038/s41419-020-03052-1 (PMC7547721; doi:10.1038/s41419-020-03052-1)

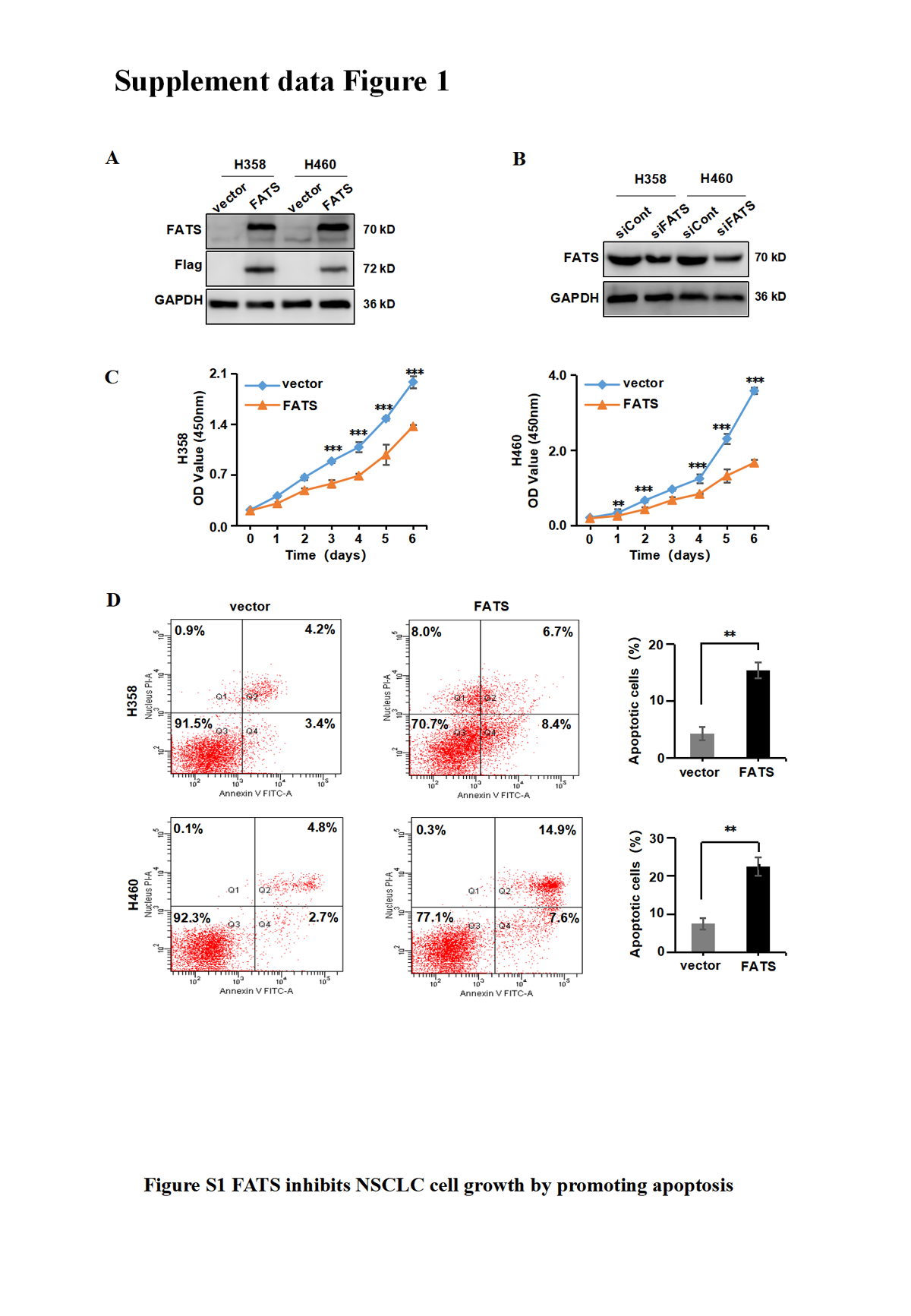

Supplement: Supplementary file 2 — revise-Figure-S01 [file 41419_2020_3052_MOESM2_ESM.tif]

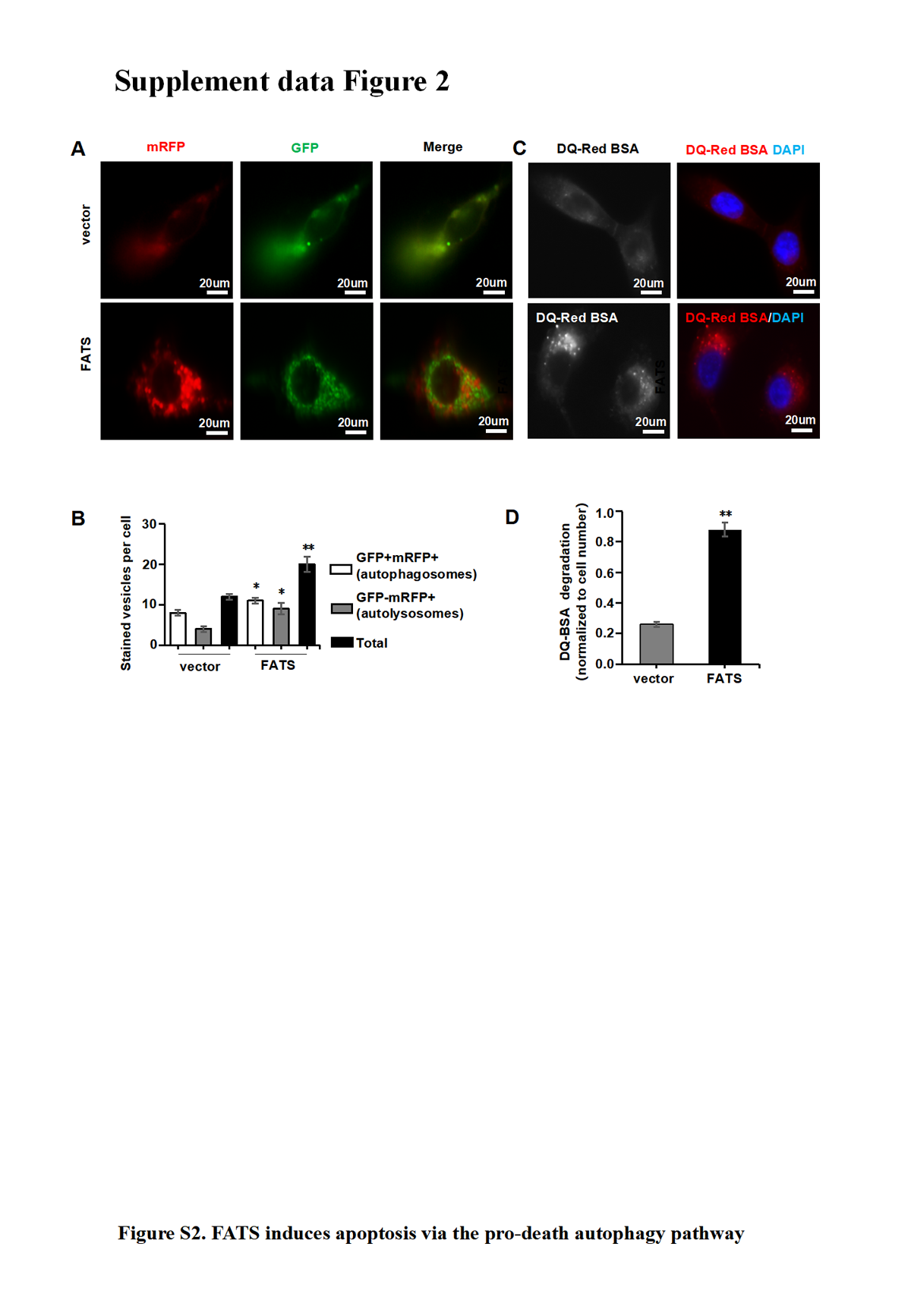

Supplement: Supplementary file 3 — revise-Figure-S02 [file 41419_2020_3052_MOESM3_ESM.tif]

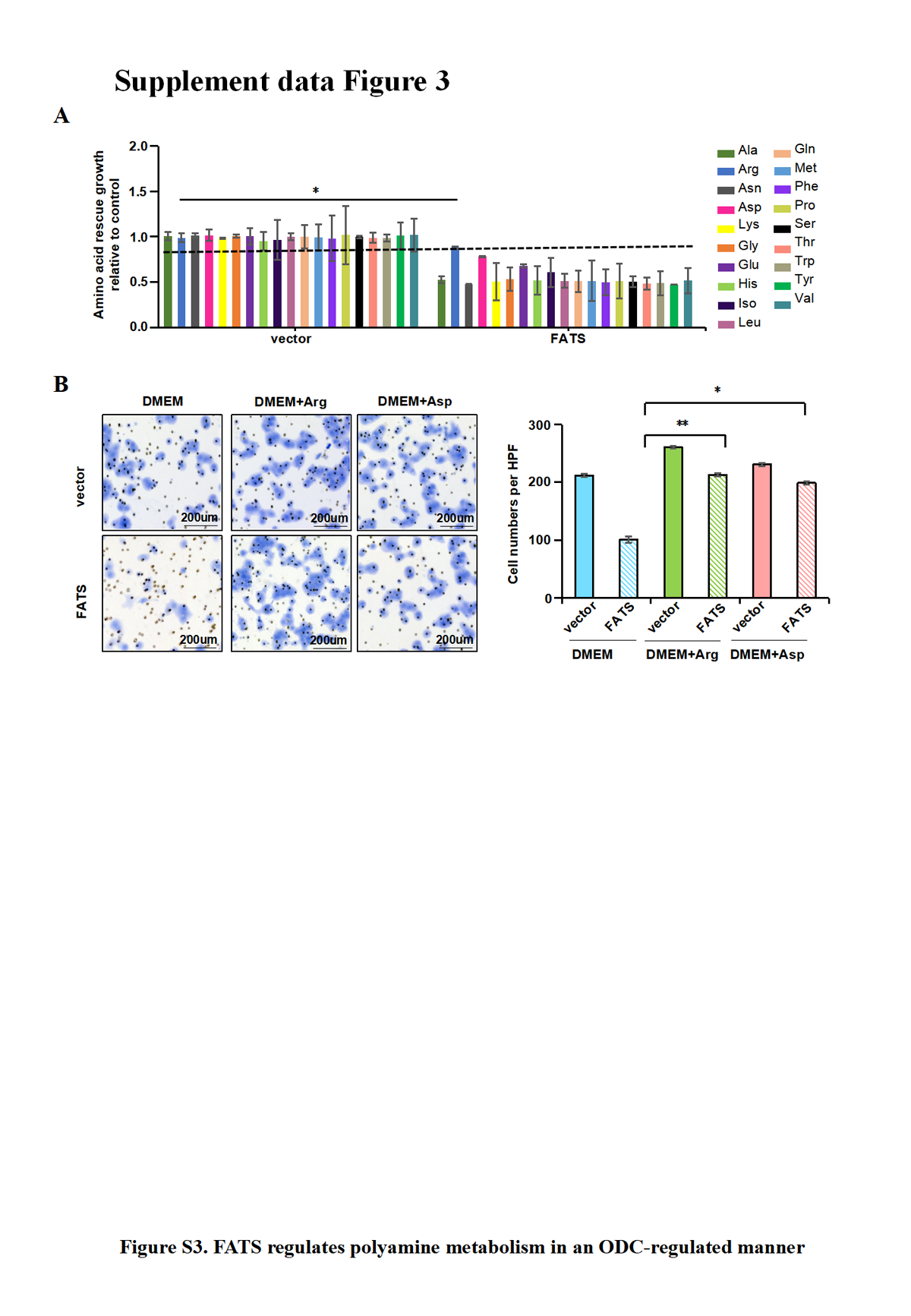

Supplement: Supplementary file 4 — revise-Figure-S03 [file 41419_2020_3052_MOESM4_ESM.tif]

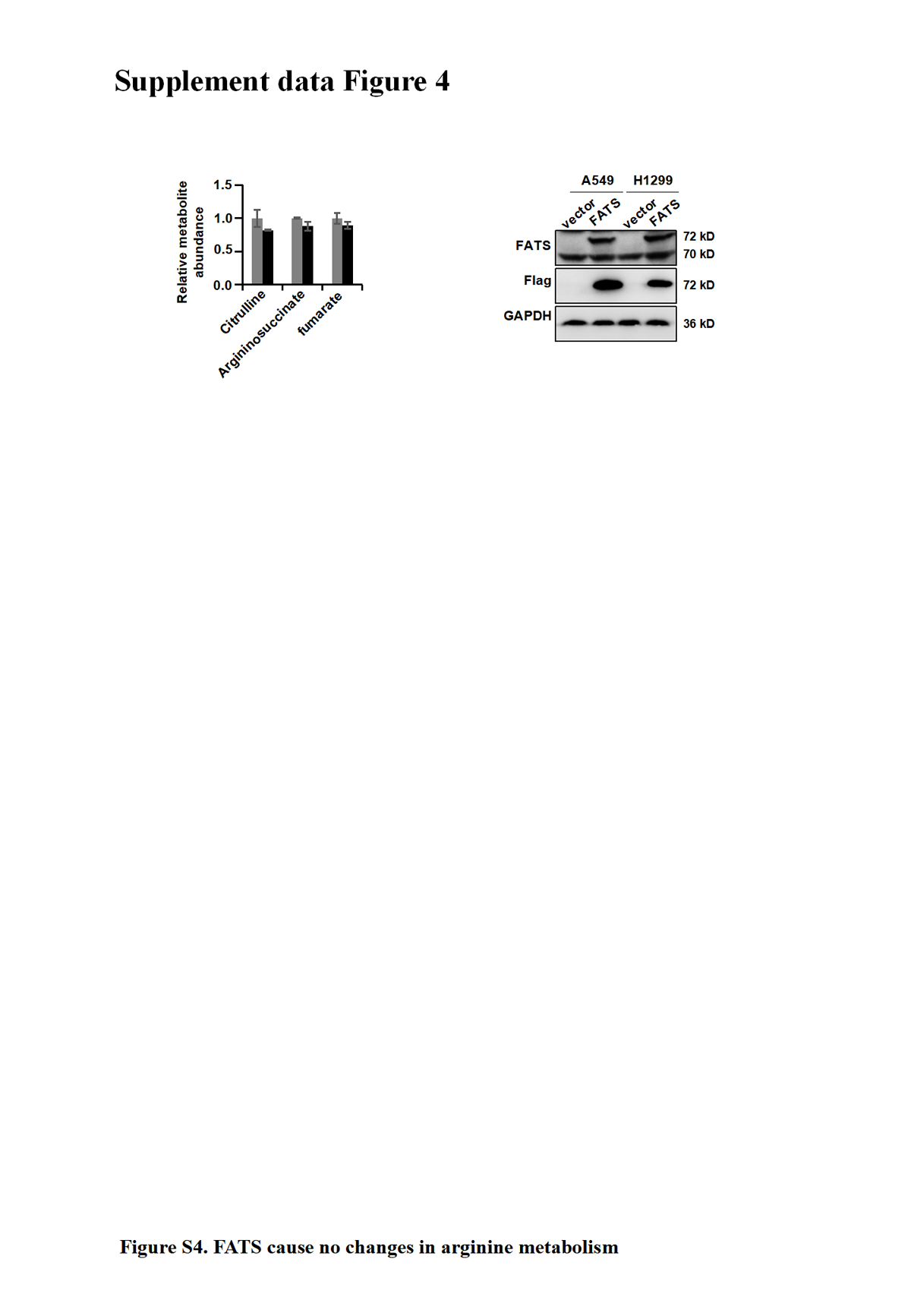

Supplement: Supplementary file 5 — revise-Figure-S04 [file 41419_2020_3052_MOESM5_ESM.tif]

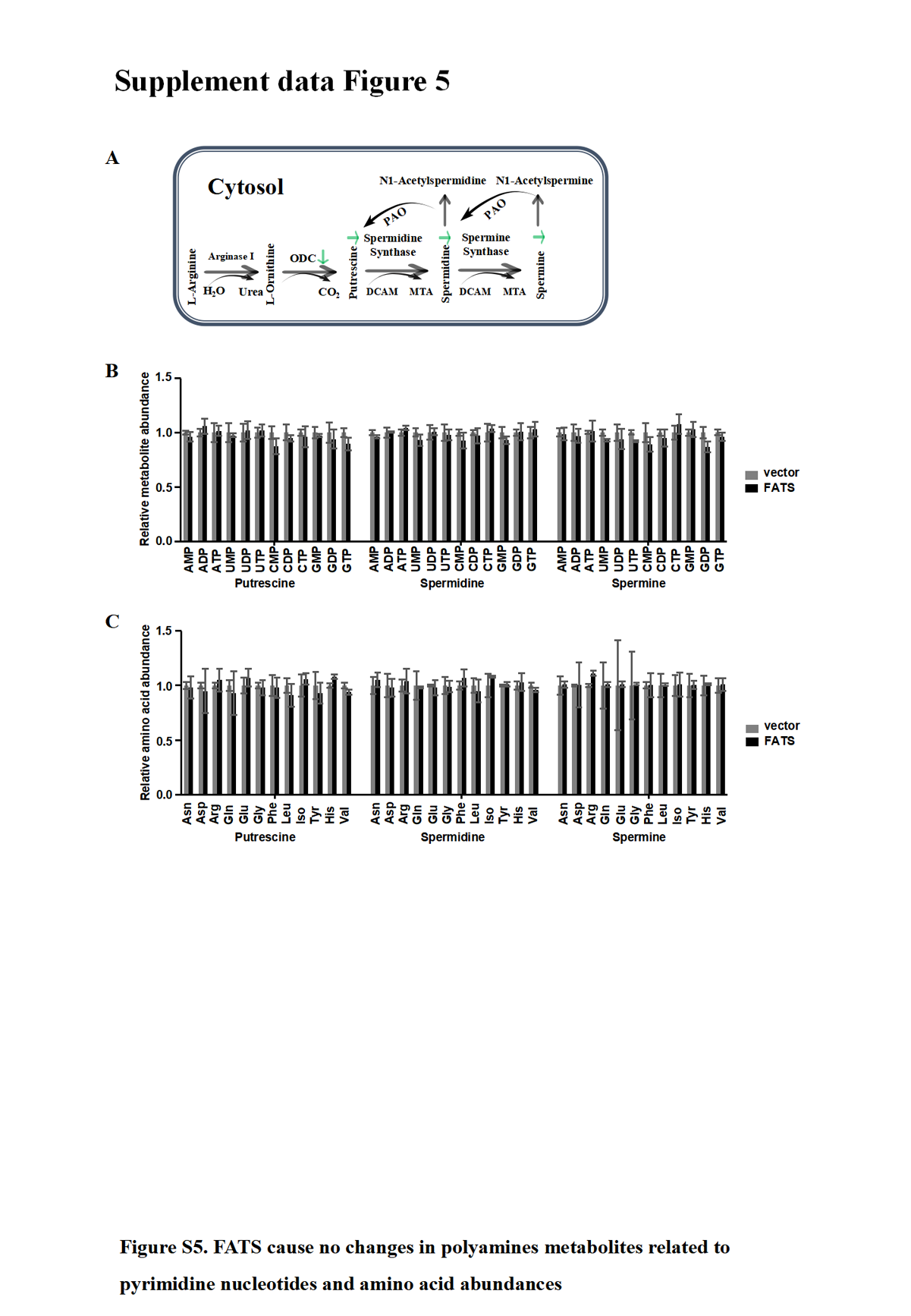

Supplement: Supplementary file 6 — revise-Figure-S05 [file 41419_2020_3052_MOESM6_ESM.tif]

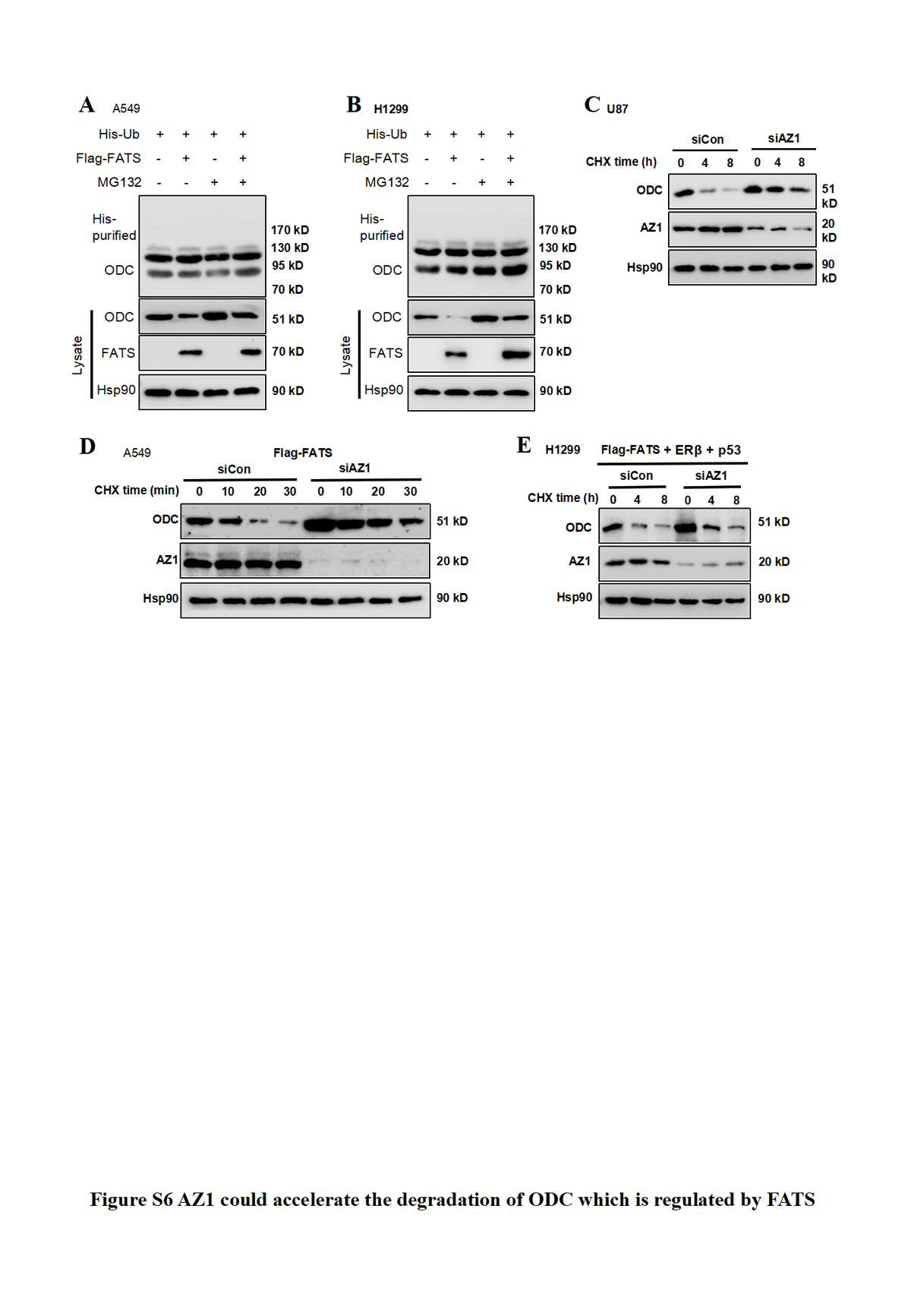

Supplement: Supplementary file 7 — revise-Figure-S06 [file 41419_2020_3052_MOESM7_ESM.tif]
