# Supplementary material for: FATS regulates polyamine biosynthesis by promoting ODC degradation in an ERβ-dependent manner in non-small-cell lung cancer
Source: Cell Death Dis. 2020 Oct 9;11(10):839. doi: 10.1038/s41419-020-03052-1 (PMC7547721; doi:10.1038/s41419-020-03052-1)
Supplement: Supplementary file 8 — siRNA [file 41419_2020_3052_MOESM8_ESM.docx]

Supplemental Table 1

| FATS | Forward | 5’-CCACAGUAGAAGAGAUCAAdTdT-3’ |
| --- | --- | --- |
|  | Reverse | 5’-UUGAUCUCUUCUACUGUGGdTdT-3’ |
| EBβ | Forward | 5’-GCCCUGCUGUGAUGAAUUAdTdT-3’ |
|  | Reverse | 5’-UAAUUCAUCACAGCAGGGCdTdT-3’ |
| p53 | Forward | 5’-GCUUCGAGAUGUUCCGAGAdTdT-3’ |
|  | Reverse | 5’-UCUCGGAACAUCUCGAAGCdTdT-3’ |
| AZ1 | Forward | 5’-ACAAGACGAGGAUUCUCAAdTdT-3’ |
|  | Reverse | 5’-UUGAGAAUCCUCGUCUUGUdTdT-3’ |
