# Supplementary material for: FATS regulates polyamine biosynthesis by promoting ODC degradation in an ERβ-dependent manner in non-small-cell lung cancer
Source: Cell Death Dis. 2020 Oct 9;11(10):839. doi: 10.1038/s41419-020-03052-1 (PMC7547721; doi:10.1038/s41419-020-03052-1)
Supplement: Supplementary file 9 — antibody list [file 41419_2020_3052_MOESM9_ESM.docx]

Supplemental Table 3

| Antibody name | Product code and manufacturer | Antibody dilution in experiments |
| --- | --- | --- |
| Flag mouse monoclonal | Cat. no. F1804, Sigma-Aldrich, USA | WB 1:5000; IP 1 µg/ml |
| FATS rabbit polyclonal | Cat. no. ab122497, Abcam, UK | WB 1:1000; IHC 1:100; IF 1:200 |
| Bak [Y164] rabbit polyclonal | Cat. no. ab32371, Abcam, UK | WB 1:1000 |
| Bax rabbit polyclonal | Cat. no. ab53154, Abcam, UK | WB 1:1000 |
| caspase3 rabbit polyclonal | Cat. no. 9665, Cell Signaling Technology, USA | WB 1:1000 |
| PARP rabbit polyclonal | Cat. no. 9542, Cell Signaling Technology, USA | WB 1:1000 |
| caspase8 rabbit polyclonal | Cat. no. 4790, Cell Signaling Technology, USA | WB 1:1000 |
| LC3A/B rabbit polyclonal | Cat. no. 4108, Cell Signaling Technology, USA | WB 1:1000 |
| [CAD](https://www.cst-c.com.cn/products/primary-antibodies/cad-d2t8h-rabbit-mab/93925?site-search-type=Products&N=4294956287&Ntt=cad&fromPage=plp) rabbit polyclonal | Cat. no. [93925](https://www.cst-c.com.cn/products/primary-antibodies/cad-d2t8h-rabbit-mab/93925?site-search-type=Products&N=4294956287&Ntt=cad&fromPage=plp), Cell Signaling Technology, USA | WB 1:500 |
| Phospho-[CAD](https://www.cst-c.com.cn/products/primary-antibodies/cad-d2t8h-rabbit-mab/93925?site-search-type=Products&N=4294956287&Ntt=cad&fromPage=plp) rabbit polyclonal | Cat. no. 70307, Cell Signaling Technology, USA | WB 1:1000 |
| p62/SQSTM1 rabbit polyclonal | Cat. no. [GTX111393](http://www.genetex.com/SQSTM1-P62-antibody-GTX111393.html), GeneTex, USA | WB 1:2500 |
| ATG5 rabbit polyclonal | Cat. no. [GTX113309](http://www.genetex.com/ATG5-antibody-GTX113309.html), GeneTex, USA | WB 1:2500 |
| Beclin1 rabbit polyclonal | Cat. no. [GTX31722](http://www.genetex.com/Beclin-1-antibody-GTX31722.html), GeneTex, USA | WB 1:2500 |
| Actin mouse monoclonal | Cat. no. [GTX11003](http://www.genetex.com/Actin-antibody-AC-40-GTX11003.html), GeneTex, USA | WB 1:5000 |
| GAPDH rabbit polyclonal | Cat. no. GTX100118, GeneTex, USA | WB 1:5000 |
| ASS1 rabbit polyclonal | Cat. no. 16210-1-AP, Proteintech, USA | WB 1:2000 |
| PKM2 rabbit polyclonal | Cat. no. 15822-1-AP, Proteintech, USA | WB 1:2500 |
| phospho-PKM2 rabbit polyclonal | Cat. no. 12822-1-AP, Proteintech, USA | WB 1:2500 |
| LDHA rabbit polyclonal | Cat. no. 19987-1-AP, Proteintech, USA | WB 1:3000 |
| LDHB rabbit polyclonal | Cat. no. 14824-1-AP, Proteintech, USA | WB 1:2500 |
| ODC goat polyclonal | Cat. no. sc-33539, Santa Cruz, USA | WB 1:2500; IP 1 µg/ml; IHC 1:100; IF 1:200 |
| ERβ rabbit polyclonal | Cat. no. sc-390243, Santa Cruz, USA | WB 1:1000; IP 1 µg/ml |
| Hsp90 rabbit polyclonal | Cat. no. sc-69703, Santa Cruz, USA | WB 1:1000 |
| AZ1 goat polyclonal | Cat. no. Ab223481, Abcam, UK | WB 1:1000 |

WB: Western blot; IHC: Immunohistochemistry; IF: Immunofluorescence; IP:Immunoprecipitation
