# Supplementary material for: FATS regulates polyamine biosynthesis by promoting ODC degradation in an ERβ-dependent manner in non-small-cell lung cancer
Source: Cell Death Dis. 2020 Oct 9;11(10):839. doi: 10.1038/s41419-020-03052-1 (PMC7547721; doi:10.1038/s41419-020-03052-1)
Supplement: Supplementary file 10 — RT-qPCR Primers [file 41419_2020_3052_MOESM10_ESM.docx]

Supplemental Table 2

| ODC | Forward | 5’-GCTCTTTGAAAACATGGGCG-3’ |
| --- | --- | --- |
|  | Reverse | 5’-GCATCCTGTTCCTCTACTTCG-3’ |
| GAPDH | Forward | 5’-GAAGGTGAAGGTCGGAGTC-3’ |
|  | Reverse | 5’-GAAGATGGTGATGGGATTTC-3’ |
